# *Bacillus subtilis* MutS Modulates RecA-Mediated DNA Strand Exchange Between Divergent DNA Sequences

**DOI:** 10.3389/fmicb.2019.00237

**Published:** 2019-02-13

**Authors:** Begoña Carrasco, Ester Serrano, Alejandro Martín-González, Fernando Moreno-Herrero, Juan C. Alonso

**Affiliations:** ^1^Department of Microbial Biotechnology, Centro Nacional de Biotecnología – Consejo Superior de Investigaciones Científicas, Madrid, Spain; ^2^Department of Macromolecular Structures, Centro Nacional de Biotecnología – Consejo Superior de Investigaciones Científicas, Madrid, Spain

**Keywords:** horizontal gene transfer, genetic variation, mismatch repair, RecA nucleoprotein filaments, MutS, SsbA

## Abstract

The efficiency of horizontal gene transfer, which contributes to acquisition and spread of antibiotic resistance and pathogenicity traits, depends on nucleotide sequence and different mismatch-repair (MMR) proteins participate in this process. To study how MutL and MutS MMR proteins regulate recombination across species boundaries, we have studied natural chromosomal transformation with DNA up to ∼23% sequence divergence. We show that *Bacillus subtilis* natural chromosomal transformation decreased logarithmically with increased sequence divergence up to 15% in wild type (*wt*) cells or in cells lacking MutS2 or mismatch repair proteins (MutL, MutS or both). Beyond 15% sequence divergence, the chromosomal transformation efficiency is ∼100-fold higher in Δ*mutS* and Δ*mutSL* than in Δ*mutS2* or wt cells. In the first phase of the biphasic curve (up to 15% sequence divergence), RecA-catalyzed DNA strand exchange contributes to the delineation of species, and in the second phase, homology-facilitated illegitimate recombination might aid in the restoration of inactivated genes. To understand how MutS modulates the integration process, we monitored DNA strand exchange reactions using a circular single-stranded DNA and a linear double-stranded DNA substrate with an internal 77-bp region with ∼16% or ∼54% sequence divergence in an otherwise homologous substrate. The former substrate delayed, whereas the latter halted RecA-mediated strand exchange. Interestingly, MutS addition overcame the heterologous barrier. We propose that MutS assists DNA strand exchange by facilitating RecA disassembly, and indirectly re-engagement with the homologous 5′-end of the linear duplex. Our data supports the idea that MutS modulates bidirectional RecA-mediated integration of divergent sequences and this is important for speciation.

## Introduction

Genetic surveys of bacterial populations show that horizontal gene transfer (HGT) is important in acquiring genetic diversity, which provides a central role in the evolution and spread of antibiotic resistance and pathogenicity traits (Doolittle, 1999; Gogarten et al., 2002). The HGT mechanism, or the non-sexual movement of genetic material, is a major source of DNA transfer between related bacteria. Indeed, up to 20% of bacterial genes are of foreign origin (Doolittle, 1999; Gogarten et al., 2002; Fraser et al., 2007). We have limited understanding as to how HGT contributes to trait variation within a species, how selection pressures affect speciation, and how recombination rates are regulated. However, it has been reported that homologous recombination, rather than the stepwise accumulation of mutations, accounts for the major sequence differences between isolates (Doolittle, 1999; Gogarten et al., 2002). Bacteria use three basic mechanisms for HGT: transduction, conjugation, and natural transformation. Generalized transduction involves virus-mediated injection of linear double-stranded (ds) DNA; chromosomal conjugation is a cell contact-dependent transfer mechanism of linear single-stranded (ss) DNA between cells and subsequent conversion to linear dsDNA. In both cases, DNA transfer is mediated by episomal proteins, and the host recombination machinery catalyzes DNA strand exchange between a linear, end-resected 3′-tailed donor duplex and the recipient duplex genome (Kowalczykowski et al., 1994; Matic et al., 1996; Fraser et al., 2007). In contrast, natural chromosomal transformation is activated through a dedicated, host-encoded transcription program (Chen and Dubnau, 2004; Kidane et al., 2012; Takeuchi et al., 2014). This cell differentiation allows internalization of linear ssDNA in the cytosol. If sufficient homology is provided, RecA-mediated bidirectional DNA strand exchange integrates the incoming linear ssDNA into the recipient circular duplex genome (Kidane et al., 2012; Carrasco et al., 2016).

DNA sequence divergence acts as an interspecies barrier to genetic recombination (reviewed in Cohan, 1994; Matic et al., 1996; Fraser et al., 2007). In eukaryotes, populations that are separated by only 2% sequence divergence are frequently unable to exchange genes (Desalle and Hunt, 1987). A greater sequence divergence (15–16%), however, is needed to reduce bacterial conjugation or natural chromosomal transformation by ∼10^3^-fold (Rayssiguier et al., 1989; Humbert et al., 1995; Zawadzki et al., 1995; Fraser et al., 2007; Carrasco et al., 2016). Unexpectedly, as little as 3% sequence divergence blocks virus-mediated transduction efficiency by ∼10^6^-fold (Zahrt et al., 1994; Zahrt and Maloy, 1997).

When donor DNA with large patches of heterology were used to transform competent *Streptococcus pneumoniae*, *Acinetobacter baylyi*, and *Pseudomonas stutzeri* cells a RecA-dependent homology-facilitated illegitimate recombination (HFIR) event was documented, albeit with low efficiency (10^−2^ – 10^−3^ relative to homologous transformation). This hybrid recombination reaction allows integration of 3- to 10-base pairs (bp) segments (de Vries and Wackernagel, 2002; Prudhomme et al., 2002; Meier and Wackernagel, 2003). Chromosomal transformation via HFIR has not been described for *Bacillus subtili*s competent cells.

The HGT mechanism also determines how the restriction modification and mismatch repair (MMR) systems act on genetic exchange between DNA substrates. In *Escherichia coli*, the key enzymes to initiate methyl-direct MMR are MutS, MutL, MutH, UvrD and Dam. After MutS-mediated mismatch recognition, it interacts with and recruits the MutL chaperone to form a MutS⋅MutL complex. MutL, at the MutS⋅MutL complex, recruits and activates the MutH nickase, and recruits the UvrD DNA helicase. MutH nicks the unmethylated strand of the hemi-methylated GATC site. The Dam methylase and MutH activities provide a signal that directs the MMR pathway (Lahue et al., 1989; Spampinato and Modrich, 2000; Acharya et al., 2003; Winkler et al., 2011). Then, different exonucleases degrade the displaced strand containing the replication error, the resulting single-stranded gap is filled-in by repair synthesis and the remaining nick is sealed by a DNA ligase (Viswanathan et al., 2001).

The absence of MutS or MutL increases by ∼10^3^-fold intergenomic recombination between *E. coli* and *Salmonella typhimurium*, with up to 16% sequence divergence, and absence of MutH or UvrD increases interspecies conjugation by only ∼50-fold (Rayssiguier et al., 1989, 1991; Matic et al., 1995; Vulic et al., 1997). It is likely that interspecies recombination in γ-proteobacteria is negatively controlled by MutS and MutL proteins rather than by the MMR system. Indeed, *E. coli* MutS and MutL control homologous recombination by aborting RecA-mediated strand exchange between divergent DNA sequences (Worth et al., 1994; Tham et al., 2016). It has been proposed that *E. coli* MutS and MutL, by binding to secondary structures of displaced ssDNA and entrapping mismatches within the heteroduplex region, impose a rotational constraint on RecA-mediated strand exchange (Tham et al., 2013).

MMR proteins are only marginally effective at preventing natural chromosomal transformation between linear donor ssDNA and recipient supercoiled dsDNA sequences with up to ∼15% divergence (Humbert et al., 1995; Majewski and Cohan, 1998; Majewski et al., 2000; Rossolillo and Albertini, 2001; Young and Ornston, 2001; Meier and Wackernagel, 2005). MMR in eukaryotes and most bacteria do not rely on a MutH- and Dam methylation-independent pathway, and MutL acts not only as a matchmaker, but also provides endonuclease activity for strand incision (Kadyrov et al., 2006; Pillon et al., 2010). The replicase processivity clamp interacts with the MutL nickase domain and licenses MutL incision for mismatch removal on the DNA strand that contains a pre-existing nick or strand discontinuity that is usually associated with the newly synthesized DNA strand (Kadyrov et al., 2006, 2007; Simmons et al., 2008; Pillon et al., 2010, 2015; Pluciennik et al., 2010; Lenhart et al., 2013b). Transient state of development of natural competence in *B. subtilis* cells halts DNA replication, while the active transcription program possibly displaces the processivity β-clamp left on DNA behind replication forks. The MMR might thus be blind to correcting mismatches during *B. subtilis* chromosomal transformation. Questions remain as to whether MMR plays a role as an anti-recombination mechanism in bacteria with natural competence, and as to which extent sequence divergence blocks interspecies chromosomal transformation. Alternatively, the MutS paralog MutS2, which lacks the mismatch binding domain, but shares two of the four MutS domains (Rossolillo and Albertini, 2001; Burby and Simmons, 2017), might negatively control interspecies recombination and therefore genetic diversity in bacteria. Indeed, *Helicobacter pylori* MutS2 inhibits the RecA-mediated DNA strand exchange reactions (Pinto et al., 2005). To define the role of MMR and MutS2 in natural chromosomal transformation we studied *B. subtilis*, the best-characterized genetic recombination system within the phylum Firmicutes. Unless otherwise stated, the indicated genes and products are of *B. subtilis* origin.

In the natural competent *B. subtilis* subpopulation, the DNA uptake machinery assembles at one of the cell poles (Chen and Dubnau, 2004; Kidane et al., 2012). The DNA uptake apparatus binds any extracellular dsDNA, linearizes it, degrades one of the strands, and internalizes the other independently of its nucleotide sequence and polarity (Kidane et al., 2012; Takeuchi et al., 2014). RecA polymerizes on the internalized linear ssDNA with the help of the two-component mediator (SsbA and RecO or DprA), then searches for an identical segment in the centrally located chromosome (Yadav et al., 2014; Carrasco et al., 2015). When a minimal efficient processing DNA segment (MEPS) is identified, RecA initiates strand invasion by forming a displacement loop (D-loop) with the recipient dsDNA (Cox, 2007; Bell and Kowalczykowski, 2016). MEPS regions of sequence identity located at either end of the donor DNA strand have an essential role when DNA sequences are divergent (Alonso et al., 1986; Shen and Huang, 1986). The MEPS necessary to allow RecA-mediated chromosomal transformation is predicted to be 25- to 35-bp (Majewski and Cohan, 1999). *In vitro*, neither *E. coli* nor *B. subtilis* RecA can promote base pairing until a 9- to 15-bp threshold of identity is reached to stabilize their interaction, and ∼26-bp permit DNA strand exchange (Hsieh et al., 1992; Yang et al., 2015).

*Bacillus subtilis* RecA, as eukaryotic Rad51 (Namsaraev and Berg, 1998; Mazin et al., 2000), can catalyze strand exchange with incoming ssDNA in either the 5′→3′ or 3′→5′ direction (Carrasco et al., 2016). *B. subtilis* RecA shows *∼*3-fold preference to initiate DNA pairing at the 3′ over the 5′ complementary end with the circular ssDNA substrate (*css*) (Carrasco et al., 2016). In the ATP⋅Mg^2+^-bound form (denoted as RecA⋅ATP), at least *B. subtilis* and *S. pneumoniae* RecA cannot catalyze DNA strand exchange (Lovett and Roberts, 1985; Steffen and Bryant, 1999; Carrasco et al., 2008) and require a two-component mediator (SsbA and DprA or RecO) (Yadav et al., 2014; Carrasco et al., 2015, 2016). In contrast, *B. subtilis* RecA⋅dATP and *E. coli* RecA⋅ATP can catalyze strand exchange in the absence of mediators (Lovett and Roberts, 1985; Steffen and Bryant, 1999; Cox, 2007; Carrasco et al., 2008; Bell and Kowalczykowski, 2016). *E. coli* RecA⋅ATP can only catalyze unidirectional DNA strand exchange (5′→3′), although RecA⋅ATPγS can polymerize in both directions onto ssDNA (Cox and Lehman, 1981; West et al., 1981; Kowalczykowski, 1991; Konforti and Davis, 1992; Bell et al., 2012).

To study how MMR proteins regulate recombination across species boundaries, we used *B. subtilis* natural chromosomal transformation. We found that the chromosomal transformation frequency decreased in a biphasic manner with increased sequence difference. Up to 15% sequence divergence the chromosomal transformation frequency decreased logarithmically in competent wild type (*wt*), Δ*mutS*2, Δ*mutL*, Δ*mutS* and Δ*mutSL* cells. The chromosomal transformation frequencies were marginally higher (∼2-fold) in the absence of MMR proteins (Δ*mutS*,Δ*mutL*, and Δ*mutSL* strains) compared to the Δ*mutS*2 and *wt* control. At 17% sequence divergence and beyond, chromosomal transformation reached a plateau in Δ*mutS* and Δ*mutSL*. In competent Δ*mutS*2 cells or wt control the transformation frequencies decline. To determine how MutS affects RecA-mediated DNA strand exchange, we monitored DNA strand exchange reactions using a circular single-stranded DNA (*css*) and linear double-stranded DNA (*lds*) substrate with an internal 77-bp region with ∼16% (homeologous DNA) or ∼54% sequence divergence (heterologous DNA) in an otherwise homologous substrate. The internal homeologous segment delayed, and the heterologous region impeded RecA-mediated DNA strand exchange. Addition of MutS apparently overcame this heterologous barrier, and nicked circular (*nc*) products were found. MutS nonetheless did not assist RecA catalysis of DNA strand exchange if the recombining 5′-end of the dsDNA was blocked by heterology. After Worth et al. (1994) and Tham et al. (2013), we propose that a heterologous region forms a blockage to RecA filament growth and RecA-mediated DNA strand exchange, and MutS bound to the barrier indirectly facilitates the spontaneous RecA nucleoprotein filament disassembly. Free RecA⋅ATP, which is able to recombine bidirectionally, might re-engage at the distal 5′ complementary end to promote DNA strand exchange in the 3′→5′ direction. Alternatively, RecA⋅ATP can initiate DNA pairing from the distal 5′ complementary end, albeit with *∼*3-fold lower efficiency (Carrasco et al., 2016), to bypass the need of the MMR proteins.

## Materials and Methods

### Strains and Plasmids

The *B. subtilis* BG1359 (*trpCE metA*5 *amyE1 rsbV*37 *xre*1 *xkd*A1 *att*^SPß^ *att*^ICE^*^Bs^*^1^ Δ*rok*) strain (Carrasco et al., 2016) was used to construct BG1393 (Δ*mut*S Δ*mutL*), BG1531 (Δ*mutL*), BG1481 (Δ*mutS*2) and BG1633 (Δ*recA*) strains. The *mutL* gene, transcribed from its native promoter, was cloned onto the middle copy vector (7 ± 2 copies/cell) to generate pCB1018. The *mutS* and *mutL* genes forms an operon. To construct the Δ*mutS* strain avoiding any negative effect in the expression of downstream *mutL* gene the pCB1018-borne *mutL* gene was introduced by natural transformation into competent BG1393 (Δ*mut*S Δ*mutL*) cells, to render BG1393 bearing pCB1018 (Δ*mutS mutL^+^*). The product of the *rpoB*482 gene, with a mutation that confers Rif^R^, from different *Bacillus* species or subspecies is functional in the recipient BG1359 cells and its isogenic derivatives (Carrasco et al., 2016). Plasmid-borne *rpoB*482 DNA, with a mutation that confers Rif^R^, from different *Bacillus* species or subspecies (pCB980-*Bsu* 168 [99.96% sequence identity, 1 mismatch], pCB981-*Bsu* W23 [97.53%, 74 mismatches], pCB982-*Bat* 1942 [91.65%, 250 mismatches], pCB983-*Bam* DSM7 [89.88% 303 mismatches], pCB984-*Bli* DSM13 [85.48%, 435 mismatches], pCB985-*Bth* MC28 [79.17%, 624 mismatches/insertion/deletion]) have been described (Carrasco et al., 2016). Plasmid-borne *rpoB*482 DNA from *B. gobiensis* FJAT4402 pCB1054-*Bgo* [83.0% sequence identity, 510 mismatches/insertion/deletion] and *B. smithii* DSM4216 pCB1056-*Bsm* [77.26%, 681 mismatches/insertion/deletion] were synthesized *in vitro*. The dG + dC content of the different *rpoB*482 DNAs was 45.3 ± 3.3%, and the sequence identity of the RpoB protein in these genes varied from 99.9% (*rpoB*482 mutation) to 88.0%. The *E. coli* 3199-bp pGEM3 Zf(+) (Promega Biotech, Spain) was used to construct the substrate for *in vitro* assays with sequence divergence.

A 77-bp DNA segment (5′-aCATGTTCAGCGGCAGCGGATAGCGGGAAAGCGGATAGCGGCAAGCGGAAAGCGGATAGCGGTAAGCGGAAGCGGTTAcatgt-3′, *lds_hom_*); a variant with 10 mismatches (5′-aCATGTTCAGTGGCAGTGGATAGTGGGAAAGTGGATAGTGGCAAGTGGAAAGTGGATAGTGGTAAGTGGAAGTGGTTAcatgt-3′, *lds_mis_*) (15.8% sequence divergence, underlined); or a variant with 42 mismatches (5′-aCATGTTTGGCGAAGGCGAATGGCGATAGGCGAAAGGCGAACGGCGATAGGCGAAGGGCGATAGGCGACGGCGACTAcatgt-3′, *lds_het_*) (54.4% sequence divergence, underlined) and their complements were joined to *Afl*II-cleaved pGEM3 Zf(+) to render a linear 3,276-bp pGEM3_hom_ (*lds_hom_*), pGEM3_mis_ (*lds_mis_*), or pGEM3*_het_* (*lds_het_*). The 3,353-bp pGEM3_*het–ins*_ plasmid contains the 77-bp heterologous sequence at the *Afl*II- and at the *Eco*RI-cleaved site. The *E. coli* XL1-blue was used to amplify the plasmid-borne *rpoB*482 DNA (pCB980-pCB985, pCB1054, pCB1056), and the substrates for DNA strand exchange (pGEM3_hom_, pGEM3_mis_, pGEM3_het_, pGEM3_het–ins_). The 5,386-nt ϕX174 and 3,276-nt pGEM3_hom_ ssDNA were purified as described (Carrasco et al., 2005).

*E. coli* BL21(DE3)[pLysS] cells bearing pCB722 *ssbA*, pCB669 *recO*, pET28a-MutS *mutS* or pET28a-MutL *mutL* gene were used to overproduce the SsbA, RecO, MutS or MutL proteins, respectively, as described (Ayora et al., 2002; Carrasco et al., 2008; Manfredi et al., 2008; Lenhart et al., 2013a). *B. subtilis* BG214 cells bearing the pBT61 *recA* gene was used to overproduce RecA (Gassel and Alonso, 1989).

### Natural Transformation

Natural competent development was carried out as described (Alonso et al., 1988). Competent *B. subtilis* cells were transformed with plasmid-borne *rpoB*482 DNA (0.1 μg/ml) of distinct *Bacilli* origin with selection for Rif^R^ (8 μg/ml). The yield of Rif^R^ transformants was corrected for DNA uptake (assayed by determination of radioactively labeled linear DNA into cells grown to competence, measured by DNase I degradation of labeled DNA), the rate of spontaneous mutations to Rif^R^ and the values obtained were normalized relative to the parental BG1359 strain, which is considered 1 (Alonso et al., 1988; Ceglowski et al., 1990).

### Spontaneous Mutation Frequency Analysis

Natural competent *B. subtilis* cells (20–25 independent stocks of competent cells) were diluted and plated onto LB plates containing 8 μg/ml rifampicin. Appropriate dilutions were plated onto LB without antibiotic, to obtain the total number of viable cells. Plates were incubated (overnight, 37°C) and CFU counted. Mutation frequencies are calculated as the number of Rif^R^ colonies/total number of cells.

### Enzymes, Reagents, Protein, and DNA Purification

All chemicals used were analytical grade. IPTG was from Calbiochem; DNA restriction enzymes and DNA ligase were from Roche, and polyethyleneimine, DTT, ATP, dATP were from Sigma. DEAE, Q- and SP-Sepharose were from GE Healthcare, hydroxyapatite was from Bio-Rad and phosphocellulose was from Whatman.

DNA concentrations were established using the molar extinction coefficients of 8,780 and 6,500 M^−1^ cm^−1^ at 260 nm for ssDNA and dsDNA, respectively, and are expressed as moles of nucleotides. [γ^32^P]-ATP was used to labeled the ends of the *Xba*I-linearized 3,276-bp pGEM3_hom_ (*lds_hom_*), pGEM3_het_ (*lds_het_*) or 3,353-bp pGEM3*_het–ins_* (*lds_het–ins_*).

Wild type SsbA (18.7 kDa), RecO (29.3 kDa), MutS (97.6 kDa), MutL (70.4 kDa), and RecA (38.0 kDa) proteins were expressed and purified as described (Carrasco et al., 2005; Manfredi et al., 2008; Yadav et al., 2012; Lenhart et al., 2013a). All proteins were purified to 98% homogeneity. Purified SsbA, RecO, RecA or MutS in the presence of 5 mM ATP and 10 mM magnesium acetate (MgOAc) lack any protease, exo- or endonuclease activity in pGEM3 Zf(+) ssDNA or dsDNA. The corresponding molar extinction coefficients for SsbA, RecO, RecA, MutS and MutL were calculated at 280 nm as 11,400; 19,600; 15,200; 64,180; and 27,850 M^−1^ cm^−1^, respectively, as described (Carrasco et al., 2005). Protein concentrations were determined using the above molar extinction coefficients. RecA is expressed as moles of monomeric, RecO, MutS and MutL as dimeric, and SsbA as tetrameric proteins. All experiments were performed in optimal RecA conditions in buffer A [50 mM Tris-HCl (pH 7.5), 1 mM DTT, 50 mM NaCl, 10 mM MgOAc, 50 μg/ml BSA, 5% glycerol]: in these conditions, a single SSB tetramer binds in its fully wrapped (SSB_65_) binding mode to ssDNA covering ∼65-nt (Shereda et al., 2008); a RecO dimer binds 30- to 40-nt (Manfredi et al., 2010), and a RecA monomer binds 3-nt (Chen et al., 2008).

### Nucleotide Hydrolysis Assays

The ssDNA-dependent ATP or dATP hydrolysis activity of RecA (or MutS) protein was assayed via a coupled spectrophotometric enzyme assay, as reported (De La Cruz et al., 2000; Yadav et al., 2012). Assays were performed in buffer A containing 5 mM ATP or dATP (30 min, 37°C), as described (Yadav et al., 2012). The order of addition of circular 3276-nt pGEM3*_hom_* ssDNA (*css_hom_*, 10 μM in nt), of purified proteins, and the concentrations of these molecules are indicated in the text. ATP or dATP hydrolysis data were converted to ADP or dADP formation and plotted as a function of time, as described (Yadav et al., 2012). The nucleotide hydrolysis rate was derived from the slope of the linear part of the curves, as reported (Hobbs et al., 2007; Yadav et al., 2012). Lag time was derived from the time intercept of a linear regression fit to the steady state portion of data in (d)ATP hydrolysis assays, as reported (Yadav et al., 2012).

### RecA-Mediated DNA Strand Exchange

The dsDNA substrate was *Kpn*I-digested to generate *lds* with the homologous (*lds_hom_*) or divergent sequence (*lds_mis_*, *lds_het_*) region at positions 424, or the *lds_het–ins_* substrate with insertion segments at positions 424 and 3260 from the 3′-end of the strand complementary to the *css*. When indicated the *lds_het_* DNA substrate, and the *lds_hom_* control, were end-labeled T4 polynucleotide kinase and [γ^32^P]-ATP, and the products were gel-purified as described (Zecchi et al., 2012).

The *css_hom_* (10 μM in nt) was preincubated with SsbA (and RecO) in buffer A containing 5 mM ATP or dATP (5 min, 37°C). Subsequently, *lds_hom_*, *lds_mis_*, *lds_het_* or *lds_het–ins_* DNA (20 μM in nt), RecA and increasing MutL or MutS concentrations (or increasing MutS and fixed MutL) were added to the reaction and incubated (60 min, 37°C or as indicated). A (d)ATP regeneration system (8 units/ml creatine phosphokinase and 8 mM phosphocreatine) was included in all recombination reactions. After incubation, samples were deproteinized and fractionated by 0.8% agarose gel electrophoresis with ethidium bromide (Ayora et al., 2002). When indicated the fractionated reaction was gel autoradiographed. The signal of the remaining linear dsDNA substrate (*lds*), and the appearance of join molecules (*jm*) intermediates and nicked circular (*nc*) products was quantified from gels using a Geldoc (Bio-Rad) system, as described (Manfredi et al., 2008). When indicated, the sum of *jm* and *nc* is shown as % recombination.

### Atomic Force Microscopy (AFM) Sample Preparation and Imaging

Products of DNA recombination reactions were diluted in buffer A ∼1,000-fold, deposited onto freshly cleaved mica, and incubated for 30 s. The surface was thoroughly washed with 3 ml of Milli-Q water and dried under nitrogen air flow (Lyubchenko et al., 2009). The dsDNA contour length and ssDNA length were determined and estimated, respectively, as described (Fuentes-Perez et al., 2013).

Images were acquired with a Nanotec (Nanotec Electrónica, Madrid, Spain) AFM using PointProbePlus tips (PPP-NCH, Nanosensors, Neuchâtel, Switzerland). AFM was operated using tapping mode for imaging in air, at room temperature. Image processing and data extraction were done with WSxM software (Horcas et al., 2007).

## Results

### Lack of MutSL Marginally Increases Chromosomal Transformation With up to ∼15% Donor–Recipient Sequence Divergence

Inactivation of *mutL* or *mutS* increases interspecies conjugation between *E. coli* and *S. typhimurium* cells by ∼10^3^-fold relative to the *wt* control (Rayssiguier et al., 1989, 1991; Matic et al., 1995). To define the contribution of MMR or of MutS2 to natural chromosomal transformation, we tested strains isogenic to BG1359, which lacks transposons and many resident prophages, with null mutations in the *mutL* (Δ*mutL*), *mutS* and *mutL* (Δ*mutSL*), *mutS*2 (Δ*mutS*2) or *recA* (Δ*recA*) genes, or in the Δ*mutSL* strain with a plasmid-borne *mutL* gene (Δ*mutS mutL^+^*).

To define the rate of spontaneous mutations to Rif^R^ cells were developed to competence and frequencies of pre-existing or spontaneous Rif^R^ mutants assessed. The frequency of pre-existing or spontaneous Rif^R^ mutants in Δ*mutS*2 was similar to the *wt* strain (4–8 × 10^−9^) (Rossolillo and Albertini, 2001; Burby and Simmons, 2017), and was slightly lower (<2-fold) in Δ*recA* cells. In the absence of *rpoB*482 DNA, the number of Rif^R^ mutants increased 70- to 90-fold (3–6 × 10^−7^) in the Δ*mutSL*, Δ*mutL*, and Δ*mutS* strains compared to *wt* control. These data, which are similar to previous reports (Rossolillo and Albertini, 2001; Pillon et al., 2015; Lenhart et al., 2016; Burby and Simmons, 2017), show that Rif^R^ mutations accumulated in the absence of MMR.

To assess chromosomal transformation frequencies, we used the plasmid-borne *rpoB*482 DNA. A single C to T transition mutation at codon 482 in the house-keeping *rpoB* gene, which encoded for the essential β subunit of RNA polymerase, confers resistance to rifampicin (Rif^R^). To measure intraspecies chromosomal transformation, we used the plasmid-borne *rpoB*482 DNA with a single mismatch (0.04% sequence divergence) in codon 482 at position 1443 (Supplementary Figures S1A,B), and recombinants were selected by the appearance of Rif^R^ colonies (Carrasco et al., 2016). For interspecies chromosomal transformation, we selected the 2997-bp *rpoB*482 DNA derived from *Bacilli* with nearly similar dG + dC content (<3.5% variation) and sequence divergence from 2.4% (*B. subtilis* W23, 74-nt mismatches) to 22.7% (*B. smithii*, 681 mismatches/insertions/deletions) (Supplementary Figures S1A,B). These *rpoB*482 DNAs encoded RpoBs with >87% identity among them (Supplementary Figure S1C).

**FIGURE 1:**
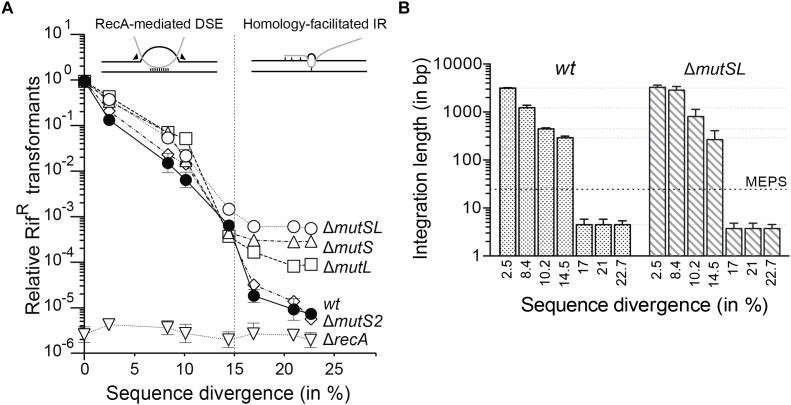
Average chromosomal transformation frequencies as a function of sequence divergence from recipient. **(A)** Top insets, black lines represent recipient chromosomal DNA and the gray line, donor linear ssDNA. The vertical dashed line denotes MEPS. Left inset, when MEPS is found, the transformation heteroduplex forms a double-ended D-loop structure. Arrowheads indicate simultaneous incision by a putative D-loop resolvase to remove the recipient strand. Right inset, when MEPS is not found, RecA anchored at the recipient dsDNA facilitates spontaneous formation of short D-loops. Plasmid-borne *rpoB*482 DNA (0.1 μg DNA/ml) from different *Bacillus* species, with the selectable Rif^R^ mutation located at position 1443, was used to transform BG1359 (*wt*, 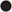) and its isogenic derivatives BG1393 (Δ*mutSL*, 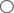), BG1531 (Δ*mutL*, 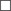), BG1393 (Δ*mutSL* plus pCB1018-borne *mutL*, Δ), BG1481 (Δ*mut*S2, 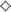), and BG1633 (Δ*recA*, ∇) competent cells. Sequence divergence values for the various *Bacillus* species were 0.04, 2.47, 8.35, 10.12, 14.52, 17.00, 20.83, and 22.74%. All data points are mean ± standard error of the mean (SEM) from three to five independent experiments. **(B)** Length of integration of donor DNA. The length of integrated DNA segments was determined by nucleotide sequence of the 2,997-bp *rpoB*482 DNA. Integration endpoints were defined by appearance of the predicted sequence of donor DNA and absence of expected mismatches in the recipient (integration midpoint). Distribution of integration endpoints up- and downstream of the Rif^R^ marker are shown for *wt* and Δ*mutSL* strains. The horizontal dashed black line denotes the MEPS and dotted gray lines denotes the small differences.

Intraspecies chromosomal transformation with *Bsu* 168 *rpoB*482 DNA (single donor–recipient mismatch) showed similar efficiency in Δ*mutS*, Δ*mutL*, Δ*mutSL* and *wt* cells (4–6 × 10^−3^; considered log 1 in Figure 1A). Chromosomal transformation frequencies were similar when *rpoB*482 DNA was replaced with *Bsu* 168 *met^+^* DNA (single donor–recipient mismatch), which confers a *met*^+^ genotype (not shown). In contrast, transformation rates were almost undetectable in the Δ*recA* strain when normalized to *wt* control (Figure 1A), and were similar to spontaneous Rif^R^ mutant frequency (5–8 × 10^−9^). Homologous chromosomal transformation frequency increased marginally (twofold to threefold) on the Δ*mutS*2 background compared with *wt* (Figure 1A), which nonetheless decreases marginally on a prophage-containing background (Burby and Simmons, 2017). This discrepancy might be attributed to background differences.

The frequency of interspecies chromosomal transformation decreased logarithmically with increased sequence divergence (to ∼15%) in all strains evaluated (Δ*mutSL*, Δ*mutS*, Δ*mutL*, Δ*mutS2*, and the *wt* control) (Figure 1A). However, these chromosomal transformation frequencies were ∼2-fold higher in the Δ*mutS*,Δ*mutL* and Δ*mutSL* strains compared to the *wt* control (Figure 1A). Similarly, lack of MutS and MutSL show a twofold to fourfold higher intergenic chromosomal transformation when compared to the *wt* control in natural competent bacteria of different phyla (Humbert et al., 1995; Majewski and Cohan, 1998; Majewski et al., 2000; Meier and Wackernagel, 2005). The frequency of appearance of Rif^R^ clones in the culture of competent Δ*recA* cells was similar to the frequency of pre-existing or spontaneous Rif^R^ mutants (Figure 1A), independently of the degree of sequence divergence.

Nucleotide sequence analyses of 10–15 Rif^R^ colonies from each genetic background showed that the heteroduplexes mainly escape the surveillance of the MMR system, the mismatches were not corrected in the sequenced clones. This observation suggests that the MMR system is easily saturated with a few mismatches (Humbert et al., 1995); alternatively, the MMR is non-functional, blind, and/or impaired in non-replicating competent cells, as proposed (Carrasco et al., 2016). The mean integration length of the *rpoB*482 DNA decreased with increased sequence divergence in Δ*mutSL*, *wt* (Figure 1B), Δ*mutS*, Δ*mutL* and Δ*mutS2* strains (not shown). At ∼15% sequence divergence the mean integration length was similar in *wt* and Δ*mutSL* strains, integration length was 130–340-bp, and 5- to 13-fold above MEPS (Figure 1B, dashed line). It is likely that chromosomal transformation with up to ∼15% DNA sequence divergence occurs via homology-directed RecA-mediated DNA strand exchange in *wt* or *mutSL* cells (Figure 1A, inset top left). Indeed, nucleotide sequence analyses of the 20–30 Rif^R^ clones appeared during transformation in the Δ*recA* context showed that all were spontaneous mutants.

### Chromosomal Transformation When Donor–Recipient Sequence Divergence Is Greater Than 15%

To analyze the limits of sequence divergence in HGT we increased sequence divergence up to ∼23% and studied the contribution of the MMR system or MutS2 to natural chromosomal transformation. When DNA sequence divergence increased from ∼15% to ∼17% in Δ*mutS*2 and *wt* cells, chromosomal transformation decreased ∼35-fold; above 17%, transformation decreased marginally (Figure 1A). In contrast, the chromosomal transformation frequencies reached a plateau in the absence of MutS and MutSL, and decreased slightly in MutL-lacking cells (Figure 1A). The frequency of interspecies chromosomal transformation with sequence divergence up to 23% was ∼40- and ∼100-fold higher in Δ*mutS* and Δ*mutSL*, respectively, compared to the *wt* strain. It is likely that the acquisition of DNA beyond 15% sequence divergence during natural transformation follows a poorly understood mechanism, and the presence of MutSL prevents chromosomal transformation.

Nucleotide sequence analyses of 20–30 Rif^R^ colonies at ∼17% and at ∼23% sequence divergence showed that 37% and 7% of the sequenced colonies, respectively, had only a single mismatch (the *rpoB*482 mutation) relative to the recipient DNA sequence. Since Rif^R^ transformants with only one mismatch cannot be distinguished from spontaneous mutants, and the transformation frequency was ∼8- and ∼3-fold greater than the spontaneous Rif^R^ mutation rate (Figure 1A), we only considered genuine transformants those inserts with at least two mismatches.

The nucleotide sequence of these transformants with ∼17% and ∼23% sequence divergence showed that the majority of genuine transformants had a mean integration length of 3–4-bp (at least two integrated non-contiguous mismatches) in the Δ*mutSL* or *wt* strains (Figure 1B), which was 8- to 10-fold below MEPS (Figure 1B, dashed line). Since the chromosomal transformation frequency in Δ*recA* cells was similar to the spontaneous mutation rate (∼5 × 10^−9^) and the chromosomal transformation efficiency at ∼23% sequence divergence in *wt* cells was ∼3-fold greater than that of Δ*recA* cells, we assumed that RecA is also needed to produce those Rif^R^ transformants with microhomologous insertions (Figure 1A, inset top right). This idea is further supported by the recent finding that integration of 3–10-bp regions, via HFIR, has been described in other natural competent bacteria of distinct phyla (*S. pneumoniae*, *A. baylyi*, and *P. stutzeri*) (de Vries and Wackernagel, 2002; Prudhomme et al., 2002; Meier and Wackernagel, 2003).

### MutS Bound to “*Mismatched DNA*” Marginally Affects RecA Nucleation and Polymerization Onto ssDNA

Lack of *E. coli* MutS or MutL increases interspecies chromosomal conjugation ∼10^3^-fold (Rayssiguier et al., 1989; Rayssiguier et al., 1991), but marginally increased (∼2-fold) interspecies *B. subtilis* chromosomal transformation (Figure 1). To define the effect of MutS (or MutSL) protein(s) on RecA-mediated DNA strand exchange we purified *B. subtilis* MutS, MutL, SsbA, RecO, and RecA and generate model DNA substrates for *in vitro* analyses. RecA- and MutS-mediated hydrolysis of ATP or dATP are employed as an indirect measure of RecA binding to ssDNA and of MutS protein binding to secondary structures with mispaired regions on the ssDNA (*mismatched DNA*). To address whether MutS⋅ATP or MutS⋅dATP interfered with RecA nucleation and filament growth ssDNA-dependent ATP and dATP hydrolysis were measured. The ssDNA-dependent ATP and dATP hydrolysis experiments were performed in conditions in which MutS specifically binds mismatched DNA (Pillon et al., 2015). In the absence of ssDNA, neither RecA nor MutS hydrolyzed ATP (Supplementary Figure S2A, inset).

As reported (Yadav et al., 2014), RecA⋅ATP nucleated and polymerized at the maximal ATP hydrolysis rate, with a k*_cat_* of 8.6 ± 0.2 min^−1^, but pre-incubation of ssDNA with stoichiometric SsbA concentrations (1 SsbA/33 nt) blocked RecA-mediated ATP hydrolysis (Supplementary Figures S2A,C). MutS hydrolyzed ATP with a k*_cat_* of 7.1 ± 0.3 min^−1^. ATP hydrolysis steady state was achieved after a 2 min lag (Supplementary Figures S2A,C). We interpret this delay as the time required by MutS to process the presence of bulges and mismatches in folded ssDNA. Similar results were observed when a genuine mismatch substrate was added (Liao et al., 2015; Lenhart et al., 2016). Pre-incubation of stoichiometric SsbA concentrations with ssDNA blocked MutS-mediated ATP hydrolysis (Supplementary Figures S2A,C). It is likely that by disassembling the “mismatched DNA,” SsbA might remove secondary structures and prevent MutS-mediated ATP hydrolysis. Since SsbA does not bind dsDNA (Yadav et al., 2012), we consider it unlikely that SsbA competes with MutS for mismatch recognition.

Combined RecA/MutS-mediated ATP hydrolysis showed a higher ATP hydrolysis rate than the isolated proteins (Supplementary Figures S2A,C), but lower than the sum of their independent activities. RecA bound to ssDNA probably promotes partial disassembly of DNA secondary structures, which in turn reduces MutS⋅ATP hydrolysis due to lesser availability of mismatched DNA (DNA secondary structures). Alternatively, MutS bound to short duplexes with bulges and mismatches might reduce RecA ability to disrupt heteroduplex structures, and passively disassemble from the ssDNA. To discriminate between these two hypotheses, we replaced ATP with dATP.

RecA-mediated dATP hydrolysis showed a biphasic curve with a ∼4 min delay for reaching maximal dATP hydrolysis (k*_cat_* of 17.9 ± 0.3 min^−1^), and SsbA pre-bound to ssDNA extended the RecA⋅dATP lag phase to ∼9 min, while maintaining maximal dATP hydrolysis rate (Supplementary Figures S2B,C) (Yadav et al., 2012). MutS-mediated dATP hydrolysis was delayed by ∼2 min in reaching the steady rate, with slightly lower efficiency (k*_cat_* 5.0 ± 0.7 min^−1^) than with ATP (Supplementary Figures S2B,C). Stoichiometric SsbA concentrations blocked MutS-mediated dATP hydrolysis (Supplementary Figures S2B,C), which implies that mispaired regions at secondary structures are crucial for MutS-mediated nucleotide hydrolysis. The RecA/MutS-mediated steady dATP hydrolysis rate was higher than that of the individual proteins, but the dATP hydrolysis rate by combined proteins was not additive. It is likely that MutS bound to duplexes with bulges and mismatches reduces the ability of RecA⋅dATP to disrupt heteroduplex structures. We nonetheless cannot rule out that RecA⋅dATP, which partially displaces SsbA from ssDNA, probably destabilizes secondary structures.

### MutS Marginally Affects RecA-Mediated DNA Strand Exchange in Reactions With Homologous Substrates

To test whether MutS or MutSL affects RecA-mediated homologous DNA strand exchange, three-strand recombination experiments were performed. During natural transformation, the internalized linear ssDNA recombines with the homologous supercoiled circular chromosome in a RecA-dependent three-strand exchange reaction. There are two constraints for this reaction *in vitro*. First, RecA⋅ATP from naturally competent bacteria requires accessory factors to catalyze DNA strand exchange (SsbA and RecO or DprA) (Yadav et al., 2014; Carrasco et al., 2015). Additionally, the three-strand exchange reaction between ∼3-kb linear ssDNA and a homologous supercoiled dsDNA substrate must be incubated with DNA topoisomerases, with a consequent reduction in recombination efficiency (Cox, 2003; Bell and Kowalczykowski, 2016). Second, RecA catalyzes strand invasion in a supercoiled duplex substrate, leading to D-loop formation, a step that is not influenced by MutS or MutL, even on mispaired substrates (Tham et al., 2016). To simplify the reaction and its subsequent analysis, we designed RecA⋅ATP-mediated three-strand exchange reactions using circular ssDNA (*css*) and linear dsDNA (*lds*) containing a 77-bp DNA homologous (*lds_hom_*), homeologous (16%, *lds_mis_*) or heterologous (54% sequence divergence, *lds_het_*) segment in an otherwise homologous substrate. In this case, the SsbA and RecO mediators are required for strand-exchange.

In the presence of the two-component SsbA-RecO mediator, *lds_hom_* and *css_hom_* DNAs (Supplementary Figure S3, open circle), optimal RecA⋅ATP concentrations catalyzed initiation of DNA recombination by pairing the homologous and complementary *lds_hom_* 3′([−] strand) with the *css_hom_* ([+] strand) DNA substrate, to yield ∼50% of recombined material in 60 min (<5% joint molecule [*jm*] intermediates and ∼46% *nc* products) (Supplementary Figure S3, lane 2). RecA-mediated DNA strand exchange is not observed when the nucleotide cofactor or when SsbA and RecO are omitted (Carrasco et al., 2015). Addition of increasing MutL, MutS or MutS plus a fixed MutL concentration reduced RecA⋅ATP-mediated strand exchange with homologous substrates by ∼2-fold (Supplementary Figure S3, lanes 3–11).

### An Internal 77-bp Region With ∼16% Sequence Divergence Delays RecA-Mediated Strand Exchange

DNA sequence divergence of 3% is reported to reduce *E. coli* RecA-mediated DNA strand exchange between circular M13 ssDNA and linear duplex fd substrates (193 mismatches) (DasGupta and Radding, 1982; Bianchi and Radding, 1983; Worth et al., 1994). Alternatively, two discrete internal regions with 8–9 mismatches (∼1-bp mismatch every ∼5-bp, and ∼20% sequence divergence) are likely to be sufficient to accumulate *jm* intermediates and reduce RecA-mediated strand exchange. Using *E. coli* purified M13 and fd DNA substrates and the MutS, MutL and RecA proteins it has been shown that RecA-mediated DNA strand exchange is reduced by the action of MutS and MutL with stops mainly at few discrete regions (Worth et al., 1994; Tham et al., 2013). MutSL bound to these heteroduplex regions and to secondary structures within the displaced ssDNA formed during strand exchange inhibit strand exchange by preventing DNA strand rotation within the recombination intermediate (Tham et al., 2013).

To analyze the effect of homeologous DNA during RecA-mediated strand exchange, we constructed a substrate that mimics recombination between viral M13 and fd. No homology on the 3′-end reduces DNA pairing (Cox and Lehman, 1981), thus the 77-bp region with sequence divergence was internal. As the length of a stable joint molecule (*jm*) intermediate is estimated to be 300- to 400-bp (Cox and Lehman, 1981), the homologous segment was replaced by a homeologous (10 mismatches, 16% sequence divergence) DNA region located internally at position 424 from the 3′-end in an otherwise identical linear duplex (*lds*) substrate, rendering the *lds_mis_* substrates.

**FIGURE 2:**
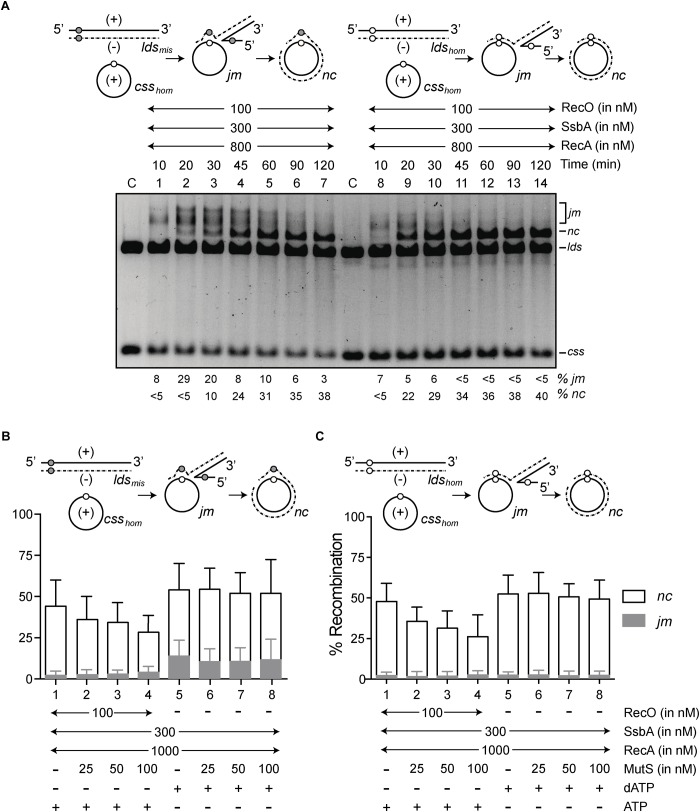
A short homeologous (77-bp segment, ∼16% sequence divergence) region on an otherwise homologous substrate (*lds_mis_*) delays RecA⋅ATP-mediated DNA strand exchange. The scheme shows the three-strand exchange reaction between *css_hom_* (+ strand) and the homeologous *lds_mis_* (filled circle) or *lds_hom_* (open circle) substrates. The 77-bp homeologous/homologous segment was restricted to an internal region toward the 3′ end. **(A)** The 3276-nt *css_hom_* DNA (10 μM in nt) was preincubated with SsbA and RecO (5 min, 37°C) in buffer A containing 5 mM ATP, followed by 3276-bp *kpn*I-linearized *lds_mis_* (lanes 1–7) or *lds_hom_* DNA (lanes 8–14) and RecA, and the reaction was incubated for various times (min) at 37°C. The reaction was separated by 0.8% agarose gel electrophoresis. Band positions correspond to substrates (*lds* and *css*), intermediates (*jm*) and product (*nc*). C denotes the DNA substrates control. The amount of recombination intermediates (*jm*) and products (*nc*) are expressed as a percentage of total substrate added. MutSL affects RecA⋅ATP rather than RecA⋅dATP-mediated DNA strand exchange. **(B,C)** The 3,277-nt homologous circular ssDNA (*css_hom_*) was pre-incubated with SsbA and RecO (5 min, 37°C) in buffer A containing 5 mM ATP or dATP. Then, RecA and increasing MutS and the 3,276-bp *lds_mis_* **(B)** or *lds_hom_* **(C)** DNA (20 μM in nt) substrate were added. The reaction was incubated (60 min, 37°C) and separated by 0.8% agarose gel electrophoresis. The amount of recombination products is expressed as a percentage of total substrate added. Quantification of intermediate/products beneath the gel shown as mean ± SEM of ≥3 independent experiments.

The *css_hom_* was preincubated with SsbA and RecO. Limiting RecA amounts and *lds_mis_* or *lds_hom_* substrate were then added and the strand exchange reaction was analyzed over time (Figure 2A). As predicted, the *lds_mis_* DNA substrate (Figure 2A, gray filled circle) would permit formation of stable DNA pairing intermediates with efficiency similar to that of the fully homologous *lds_hom_* and *css_hom_* substrates (Figure 2B, open circle) within the first 10 min (Figure 2A, lanes 1 and 8). At min 20, *jm* intermediates made up ∼30% of the *css_hom_* and *lds_mis_* substrates, and traces of final *nc* products (∼3%) were detected, although ∼22% of the *css_hom_* and *lds_hom_* substrates were converted to the final *nc* products (Figure 2A, lanes 2 and 9). Accumulation of *nc* products was nearly complete by ∼50 min with the *lds_mis_* substrate, but RecA⋅ATP yielded the maximal amount of *nc* products in ∼25 min with the *lds_hom_* substrate (Figure 2A). We show that the barrier formed by the mismatched region (77 bp with ∼16% sequence divergence) delayed RecA-mediated strand exchange. The final extent of recombination, however, was similar at ∼60 min even at limiting RecA concentrations (Figure 2A, compare lanes 5 and 12). Reaction kinetics is similar in the presence of *E. coli* RecA and M13 *css* and fd *lds* DNA substrates (Fabisiewicz and Worth, 2001).

### MutS Marginally Reduces RecA⋅ATP-Mediated DNA Strand Exchange

To test whether increasing concentrations of MutS impairs RecA-mediated homeologous DNA strand exchange or the total accumulation of *nc* products, the *lds_mis_* DNA substrate was compared to the *lds_hom_* DNA. In the presence of RecO and SsbA, increasing concentrations of MutS marginally impaired RecA⋅ATP-mediated DNA strand exchange or the total accumulation of *nc* products, with the homeologous DNA substrates when compared to absence of MutS (Figure 2B, conditions 2–4 vs. 1). Similar results were observed when the *lds_mis_* DNA was replaced by the *lds_hom_* DNA and MutS or MutS and MutL were present (Figure 2C, conditions 2–4 vs. 1 and Supplementary Figure S3, lanes 9–11 vs. 2).

RecA⋅dATP binds ssDNA with high affinity and cooperativity, displaces secondary structures and SsbA from ssDNA and can catalyze DNA strand exchange in the absence of the RecO mediator (Manfredi et al., 2008). To test whether RecA⋅dATP can overcome the negative effect of MutS, we carried out DNA strand exchange reactions at increasing MutS concentrations and in the absence of RecO (Figure 2B, conditions 5–8). The proportion of *jm* intermediates increased (9–12%) with the homeologous substrates relative to the homologous substrate (Figure 2B,C, condition 5). Increasing MutS concentrations did not affect the accumulation of final recombinant products (49–52%) with the homeologous or homologous substrates when compared to its absence (Figure 2B,C, conditions 6–8 vs. 5).

These data suggest that (i) a single internal 77-bp region with ∼16% sequence divergence moderately delays recombination, but it does not impair RecA⋅ATP- or RecA⋅dATP-mediated strand exchange in a 60 min reaction; and (ii) MutS reduces RecA⋅ATP-mediated DNA strand exchange between homologous or homeologous DNA substrates to similar extent in a 60 min reaction when compared with absence of MutS (Figure 2B,C, conditions 2–4 vs. 1). In contrast, *E. coli* MutS inhibits RecA⋅ATP-mediated branch migration between M13 *css* and fd *lds* DNA substrates with a total sequence divergence of ∼3% (or two discrete segments with up to ∼20% sequence divergence) (Worth et al., 1994; Tham et al., 2013).

### MutS Assists RecA in Overcoming an Internal 77-bp Heterologous Barrier

**FIGURE 3:**
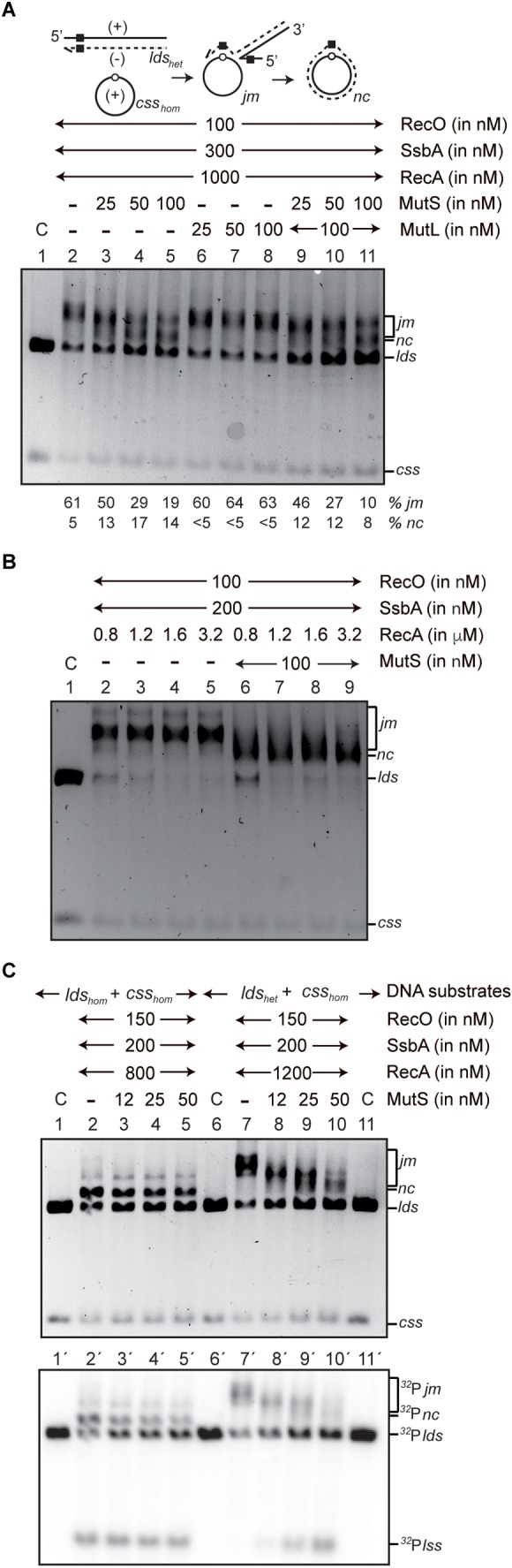
RecA⋅ATP-mediated recombination of a short heterologous (77-bp, ∼54% sequence divergence) substrate (*lds_het_*) in the presence of MutSL. **(A)** Scheme of the reaction between *css_hom_* and the heterologous *lds_het_* (black square) substrates. The 77-bp heterologous segment was restricted to an internal region toward the 3′ end. The *css_hom_* DNA (10 μM in nt) was preincubated with SsbA and RecO (5 min, 37°C) in buffer A with 5 mM ATP, followed by RecA, (MutS, MutL or MutSL) and the *lds_het_* substrate, and incubated (60 min, 37°C). Band positions correspond to substrates as in Figure 2. The minus (–) symbol denotes absence of MutS or MutL. C denotes the DNA substrates control. The percentage of *jm* intermediates and *nc* products are shown beneath the gel. Results shown as mean ± 5% SEM of ≥3 independent experiments. **(B)** Experiments with dATP and increasing RecA concentrations. The 3276-nt *css_hom_* DNA was preincubated with SsbA and RecO (5 min, 37°C) in buffer A containing 5 mM dATP. The *lds_het_* substrate, increasing RecA concentrations and, where indicated, a fixed MutS concentration were then added and incubated (60 min, 37°C). **(C)** The *css_hom_* DNA (10 μM in nt) was preincubated with SsbA and RecO (5 min, 37°C) in buffer A with 5 mM ATP, followed by RecA, the [γ^32^P]-*lds_hom_* (lanes 2–5) or [γ^32^P]-*lds_het_* lanes 7–10 substrate, in the presence or absence of increasing MutS concentrations, and incubated (60 min, 37°C). Band positions correspond to substrates as in Figure 2. The minus (–) symbol denotes absence of MutS.

*E. coli* RecA cannot catalyze DNA strand exchange with viral ϕX174 vs. G4 DNAs with an overall sequence divergence of 30% (with discrete segments with up to 55%, defining heterology) (DasGupta and Radding, 1982). To analyze the effect of heterologous DNA during RecA-mediated strand exchange, we constructed a substrate with a 77-bp heterologous region, but with identical dC:dG content, by inverting the 77-bp region. This segment (42 mismatches, ∼54% sequence divergence) was restricted to an internal region at position 424 from the 3′-end (*lds_het_*, heterologous DNA) in an otherwise homologous substrate (Figure 3A, black filled square).

In the presence of SsbA and RecO, RecA⋅ATP initiated DNA pairing between *css_hom_* and *lds_het_* at the homologous segment, leading to accumulation of *jm* intermediates, and *nc* products were barely detected in a 60-min reaction (Figure 3A, lane 2). This observation is consistent with heteroduplex joints stopped at the heterologous region. Results were similar in experiments with increasing MutL concentrations (Figure 3A, lanes 6–8), suggesting that the latent MutL endonuclease activity (Kadyrov et al., 2006, 2007; Pillon et al., 2010; Pluciennik et al., 2010) did not contribute to removing the heterologous DNA barrier.

When MutS was present, *nc* products were observed, suggesting an apparent resolution of the recombination barrier (Figure 3A, lanes 3–5). Accumulation of *nc* products was also observed with fixed MutL and increasing MutS concentrations (Figure 3A, lanes 9–11), suggesting that MutS or something in the MutS preparation is necessary and sufficient to remove the heterologous DNA barrier for RecA-mediated DNA strand exchange.

Several possibilities might explain this MutS-mediated alleviation of the apparent RecA heterologous barrier for genetic recombination. First, RecA that stalls at the heterologous duplex might revert intermediates to the original substrates, but MutS assembled at the heteroduplex region might stabilize it to allow RecA bypass of the heterology. Second, a nuclease contaminant in the MutS preparation could remove the mispaired region. Third, the MutS clamp-like structure might facilitate RecA reinitiation beyond the heterologous region. Finally, MutS diffusion along the duplex DNA might cap RecA polymerization, and thus indirectly cause RecA disassembly. Disassembled RecA might initiate DNA strand exchange from the 5′-end and catalyze recombination in the 3′→5′ direction, as described (Carrasco et al., 2016).

### RecA Overcomes the Heterologous Barrier in the Presence of MutS

To test whether MutS helps RecA to bypass or reinitiate strand exchange beyond the region of heterology, we sought optimal RecA conditions by replacing ATP with dATP and by increasing RecA concentrations (1 RecA/12- to 3-nt) (Figure 3B). The *css_hom_* DNA substrate was preincubated with RecO and SsbA. Increasing RecA⋅dATP concentrations and the *lds_het_* DNA were added and the reaction incubated (60 min, 37°C). RecA⋅dATP initiated DNA pairing and converted >95% of the *lds_het_* substrate into *jm* intermediates, although no *nc* products were detected (Figure 3B, lanes 2–5). Regardless of the RecA concentration used, RecA⋅dATP did not overcome the heterologous barrier, and the reaction probably halted at the heterologous region rather than reverting the *jm* intermediates to the original substrates. It is likely that: (i) spontaneous RecA disassembly from the nucleoprotein filament is slow under our experimental conditions; and (ii) excess of RecA and the presence of SsbA should titrate the displaced strand as competitor for pairing.

Our data showed that addition of MutS to the reaction mixture was sufficient to apparently stimulate the accumulation of a novel band that migrate as a *nc* product (Figure 3B, lanes 6–9). This suggests that the strand exchange reaction starts at the 3′ complementary end and halts at the heterologous barrier. To overcome this barrier and start the DNA strand exchange at the 5′ complementary end (see below) it requires an accessory factor (MutS) rather than passive RecA disassembly (see below). Alternatively, a contaminate nuclease in the MutS preparation might degrade the heterologous barrier or the displaced strand during RecA-mediated DNA strand exchange and stabilize the heteroduplex joint by removing the competitor strand for pairing as reported (Corrette-Bennett and Lovett, 1995). To the latter hypothesis the *lds_hom_* and *lds_het_* substrates were [γ^32^P]-end-labeled and gel purified. The *css_hom_* DNA substrate was preincubated with RecO and SsbA, and then RecA⋅ATP and [γ^32^P]-*lds_hom_* or -*lds_het_* DNA was added, and the reaction incubated (60 min, 37°C) (Figure 3C). In the presence of homology, limiting RecA⋅ATP generated *jm* intermediates and *nc* products between the *css_hom_* and the *lds_hom_* DNA (Figure 3C, lane 2) and [γ^32^P]-*jm* intermediates and [γ^32^P]-*nc* and [γ^32^P]-linear ssDNA (*lss*) products observed when the reaction was autoradiographed (Figure 3C, lane 2′). In the presence of increasing MutS concentrations, the accumulation of the *nc* products was slightly reduced as described above (Figure 3C, lanes 3–5 vs. 2) and [γ^32^P]-*nc* and [γ^32^P]-*lss* products were observed upon autoradiographed (Figure 3C, lane 2′–5′). No apparent degradation of the [γ^32^P]-*lds_hom_* substrate was observed discarding the possibility of the presence of a nuclease contaminating the MutS preparation.

For the heterologous substrate, 25–27% of the [γ^32^P]-*lds_het_* and *css_hom_* DNA substrates were converted into slow-moving [γ^32^P]-*jm* intermediates, but products with fast mobility (e.g., [γ^32^P]-*nc* and [γ^32^P]-*lss*) were not detected in the absence of MutS (Figure 3C, lanes 7 and 7′). Increasing concentrations of MutS facilitated the conversion of slow-moving [γ^32^P]-*jm* intermediates onto products with a mobility expected for [γ^32^P]-*nc* (Figure 3C, lanes 8–10) concomitant with the increase of [γ^32^P]-*lss* products when the reactions were autoradiographed (Figure 3C, lanes 8′–10′). The conversion of the [γ^32^P]-*lds_hom_* substrate onto a slow-moving [γ^32^P]-*jm* intermediates and [γ^32^P]-*nc* and [γ^32^P]-*lss* products discarding the possibility of the presence of a nuclease contaminating the MutS preparation.

**FIGURE 4:**
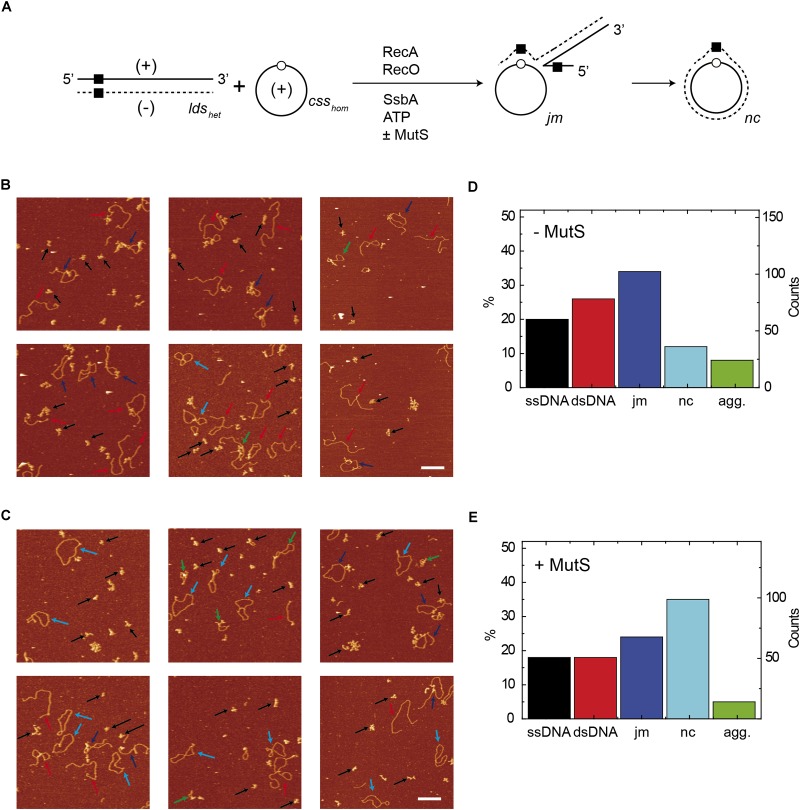
Quantification of species from AFM images of reactions containing *lds_het_* and *css_hom_* DNA. **(A)** Scheme of the reaction between *css_hom_* (open circle) and the *lds_het_* (black square) substrates, showing the *jm* intermediates, and the final *nc* product. The 3276-nt *css_hom_* DNA (10 μM in nt) was preincubated with SsbA, RecO, and RecA (60 min, 37°C) in buffer A containing 5 mM ATP, followed by linearized 3276-bp *lds_het_* DNA, alone **(B)** or with MutS **(C)**. Quantification of the different DNA species in the absence of MutS (*n* = 284 molecules) **(D)** or in the presence of MutS (*n* = 301 molecules) **(E)**. Quantification only includes dsDNA molecules with the original length and ssDNA molecules with a volume corresponding to the original length. Agg, *lds_het_* DNA with *css_hom_* at both ends or more complex aggregates. Arrows point to examples of the different species (black: ssDNA; red: dsDNA; dark blue: joint molecules; light blue: nicked circular; green: Agg).

### Genuine RecA-Mediated *nc* Products Are Formed in the Presence of MutS

To confirm that *bona fide nc* molecules are obtained in heterologous DNA strand-exchange reactions, we imaged products of these reactions using atomic force microscopy (AFM). The *css_hom_* DNA was preincubated with SsbA and RecO, and then RecA and *lds_het_* DNA were added and incubated (60 min, 37°C). Then proteins were removed from the reaction and DNA products absorbed onto mica disks. AFM images showed different forms of *jm* intermediates and *nc* products as depicted in Figure 4A. RecA⋅ATP converted ∼34% of the substrates to *jm* intermediates in 60 min (Figure 4B and quantified in Figure 4D). The rest were classified as ssDNA or dsDNA and ∼8% of the molecules formed complex structures (aggregates, Agg). From the contour of the *jm*s it could be deduced that ∼19% of them had overcame the region of heterology, suggesting that strand exchange initiated from the 5′-complementary end or spontaneous migration upon deproteinization might account for those molecules. *Circa* 12% of the substrates with a 77-bp heterologous region were converted to the final *nc* product with the anticipated contour of full-length molecules (Figure 4B,D [−MutS], *n* = 301, pointed by light blue arrows). The contribution of spontaneous branch migration upon deproteinization cannot be ruled out.

MutS addition to the reaction reduced the total amount of *jm* intermediates formed (∼24%), and notably increased the RecA⋅ATP-mediated *nc* products (∼35%) with the anticipated contour length of full-length *nc* products (Figure 4C,E [+MutS], *n* = 282 pointed by light blue arrows). These experiments revealed that in the presence of MutS the proportion of *nc* products increased ∼3-fold when compared to the absence of MutS (Figure 4D,E). It is likely that in the presence of MutS, RecA-mediated strand exchange of *css_hom_* with the complementary 5′-end of *lds_het_* in the 3′→5′ direction might explain the *nc* products found (Figure 4C,E). This is consistent with the observation that *B. subtilis* RecA can catalyze bidirectional DNA strand exchange with *∼*3-fold preference to initiate DNA pairing at the 3′ complementary end with the *css* substrate (Carrasco et al., 2016).

Control experiments with only *lds_het_* and *css_hom_* (Supplementary Figure S4A) showed homogeneous preparations of *css_hom_*, *css_hom_* and *lds_het_* or *lds_het_* (Supplementary Figures S4B–D) with the predicted contour length of *lds_het_* and *css_hom_* volume (Supplementary Figures S4E,F).

### Heterology at the 5′-End Prevents MutS-Assisted Recombination

**FIGURE 5:**
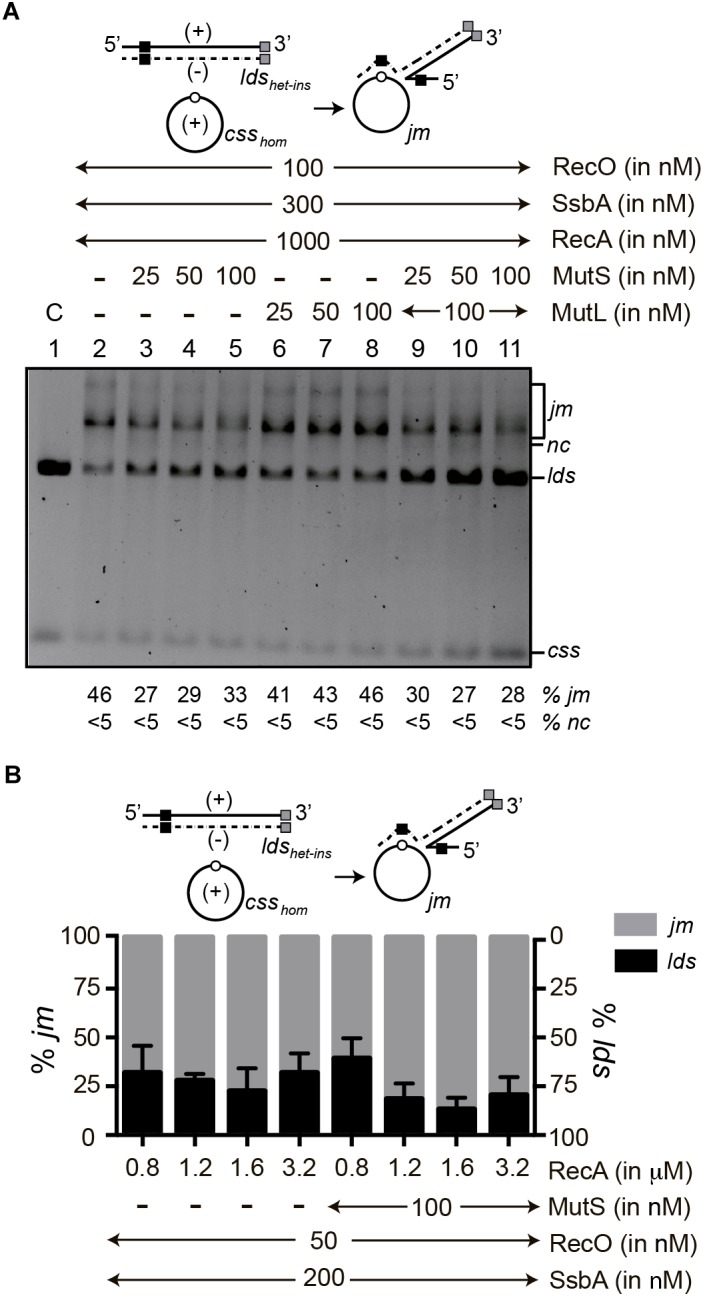
RecA⋅ATP-mediated strand exchange with short internal heterology and at the 5′-end in the presence of MutSL. **(A)** Scheme of the reaction between *css_hom_* and the *lds_het–ins_* substrate with internal region toward the 3′ end and at the 5′-end (black and gray squares, respectively). The *css_hom_* DNA (10 μM in nt) was preincubated with SsbA and RecO (5 min, 37°C) in buffer A containing 5 mM ATP, after which RecA, (MutS, MutL or MutSL) and the *lds_het–ins_* substrate were added and incubated (60 min, 37°C). The percentage of *jm* intermediates and *nc* products are shown beneath the gel. Lane 1, the *css* and *lds* substrates (termed C). Results shown as the mean ±5% SEM of ≥3 independent experiments. **(B)** Experiments with dATP and increasing RecA concentrations. The 3276-nt *css_hom_* DNA was preincubated with SsbA and RecO (5 min, 37°C) in buffer A containing 5 mM dATP, followed by the 3353-bp *lds_het–ins_* substrate, increasing RecA concentrations and, where indicated, a fixed MutS concentration, and incubated (60 min, 37°C). The reaction was separated by gel electrophoresis and quantified. The left-hand side bar denotes the fraction of *jm* intermediates (gray bar) and the right-hand side scale denotes the fraction of unreactive *lds_het–ins_* substrate (black bars). The minus (–) symbol indicates lack of MutS or MutL.

We hypothesized that MutS bound to the heterologous DNA might halt RecA filament growth and facilitate RecA passive disassembly from the nucleoprotein filament. This, could then facilitate RecA polymerization from the 5′-end. If heterology is placed at the 5′-end RecA-mediated strand exchange in the 3′→5′ direction should be blocked, and MutS should not assist RecA-mediated DNA strand exchange. To test this concept, we constructed a substrate containing a second 77-bp heterologous region at the 5′-end (Figure 5A, black and gray squares), termed the *lds_het–ins_* substrate (heterology at positions 424 and 3271).

RecO and SsbA were preincubated with *css_hom_* DNA, after which *lds_het–ins_* DNA and RecA⋅ATP were added and incubated (60 min, 37°C). RecA⋅ATP initiated DNA pairing at the homologous complementary 3′-end and accumulation of intermediates/product that ran with a mobility expected for *jm* intermediates was observed. Material that ran with a mobility compatible with *nc* products was not observed (Figure 5A, lane 2). Addition of MutS, MutL or MutSL did not lead to *nc* product accumulation (Figure 5A, lanes 3–5, 6–8, and 9–11). No apparent degradation of the *lds_het–ins_* substrate to overcame the heterologous barrier was observed.

To confirm that MutS assembled at the heteroduplex segment does not facilitate RecA reinitiation beyond the heterologous region, we replaced ATP with dATP and increased RecA concentrations. The *css_hom_* substrate was preincubated with RecO and SsbA, after which *lds_het–ins_* and increasing RecA concentrations were added and incubated (60 min, 37°C) (Figure 5B). Independently of the RecA concentration (1 RecA/12-3 nt), MutS addition did not convert *jm* intermediates to *nc* products (Figure 5A,B). In the presence of MutS, RecA⋅dATP (1 RecA/6 nt) converted ∼80% of the substrate onto *jm* intermediates (Figure 5B, left hand side scale), with ∼20% the substrate remaining as *lds* substrate (Figure 5B, right hand side scale).

**FIGURE 6:**
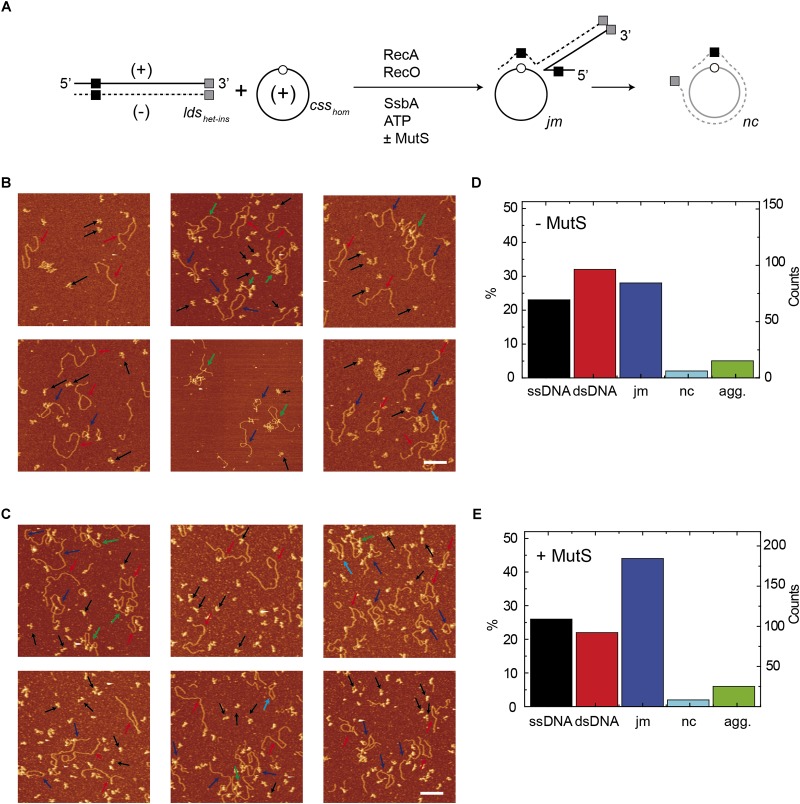
Quantification of species from AFM images of reactions containing *lds_het–ins_* and *css_hom_* DNA. **(A)** Scheme of RecA-mediated recombination reaction between *css_hom_* (open circle) and *lds_het–ins_* (internal black square and a gray square at 5′-end) substrates, showing the *jm* intermediates and the hypothetical final *nc* product (in gray). The 3276-nt *css_hom_* DNA (10 μM in nt) was preincubated with SsbA, RecO and RecA (60 min, 37°C) in buffer A with 5 mM ATP. Then, the 3353-bp *lds_het–ins_* DNA was added without **(B)** or with MutS **(C)**. Quantification of the different DNA species observed in the absence of MutS (*n* = 293 molecules) **(D)** or in the presence of MutS (*n* = 419 molecules) **(E)**. Quantification only includes dsDNA molecules with the original length and ssDNA molecules with a volume corresponding to the original length. Agg, *css_hom_* with *lds_het–ins_* DNA at one end and internal regions or more complex aggregates. Arrows point to examples of the different species (black: ssDNA; red: dsDNA; light blue: nicked circular; green: Agg).

The intermediates of strand-exchange reactions using these new substrates were imaged and analyzed using AFM. The *css_hom_* substrate was preincubated with SsbA and RecO, followed by RecA⋅ATP (±MutS) and *lds_het–ins_* DNA and incubated (60 min, 37°C) (Figure 6A,B). The contour length of the protein-free *lds_het–ins_* and *css_hom_* intermediates showed that the *jm* intermediates mainly halted at the 424-bp position (heterologous region) made up the largest fraction (∼29%). Spontaneous branch migration upon deproteinization might account for ∼13% of these *jm*s whose contour had overcame the region of heterology. Importantly, *nc* products were rarely observed (<3%) (Figure 6B,D [−MutS], *n* = 293), suggesting that RecA-mediated DNA strand exchange initiated from the 3′-complementary end and in the 5′→3′ direction could not render *nc* product.

Addition of MutS to the reaction decreased the *lds_het–ins_* substrate and increased the total amount of *jm* intermediates (∼45%), with ∼16% of them overcoming the heterologous region. This suggests spontaneous branch migration, but *nc* products were rarely observed upon deproteinization (Figure 6C,E [+MutS], *n* = 419). All together, these data suggest MutS cannot assist RecA to produce *nc* if entry from the 5′ complementary end is blocked by heterology. Then, RecA-mediated DNA strand exchange is halted at the internal heterologous sequence leading to the accumulation of *jm* intermediates.

## Discussion

This study describes the molecular keys to specialization in natural competent *B. subtilis*. The results indicate that interspecies chromosomal transformation frequency decreased in a biphasic mode with increased sequence divergence. We infer that interspecies chromosomal transformation with up to 15% provides a ∼10^3^-fold barrier to HGT. This HGT event occurs via homology-directed RecA-mediated DNA strand exchange. Beyond 15% and up to ∼23% sequence divergence genuine rare transformants, which might contribute to facilitate the restoration of inactivated genes without compromising its speciation, were observed. Finally, the analysis of the integrated DNA with ∼23% sequence divergence revealed that a very large fraction of sequenced Rif^R^ colonies did not differ from the spontaneous mutants (24 of 26 sequenced events), suggesting that beyond 23% sequence divergence HFIR should be not operative at least in non-replicating haploid competent *B. subtilis* cells.

### Homology-Directed RecA-Mediated DNA Strand Exchange

At up to 15% sequence divergence, the frequency of interspecies chromosomal transformation decreased logarithmically in the Δ*mutSL*, Δ*mutS*2 and *wt* strains compared to intraspecies transformation, but it was blocked in competent Δ*recA* cells (Figure 1A). A quantitative analysis of these data suggests that lack of MutS (or MutSL) makes marginal contribution (∼2-fold) to RecA-mediated chromosomal transformation. A similar minor effect of absence of the MMR pathway in interspecies chromosomal transformation is described for other natural competent bacteria of distinct phyla (Humbert et al., 1995; Majewski and Cohan, 1998; Majewski et al., 2000; Young and Ornston, 2001; Meier and Wackernagel, 2005). In this work, we show that the DNA mismatches of the integrated DNA onto the non-replicating haploid chromosome were neither corrected by the *B. subtilis* MMR system nor with the poorly characterized MutS2 protein. The mean integration length of DNA with ∼15% sequence divergence was 5- to 14-fold above MEPS in *wt* and Δ*mutSL* cells, but with ∼17% sequence divergence was 8- to 10-fold below MEPS (Figure 1B). These results are compatible with RecA-mediated DNA strand exchange, and suggest that between 15 and 17% sequence divergence might lay the limit in the delineation of a species.

Interspecies conjugation with up to 16% sequence divergence, however, increases ∼10^3^-fold in the *mutS* or *mutL* context in γ-proteobacteria when compared to the *wt* control (Rayssiguier et al., 1989, 1991; Vulic et al., 1997). To understand why MutS and MutL only provide a marginal barrier to chromosomal transformation (∼2-fold) up to 15% sequence divergence (Figure 1A), we performed *in vitro* studies using a simplified experimental approach; a three-strand exchange reaction with a single internal 77-bp segment with different degree of sequence divergence, and purified *B. subtilis* proteins.

The 77-bp segment with ∼16% homeology moderately delayed RecA⋅ATP-mediated DNA strand exchange between *css_hom_* and *lds_mis_* without affecting the total recombination yield in a 60 min reaction when compared to homologous DNA substrates (Figure 2A, lanes 1–7 vs. 8–14). Whereas, two discrete regions with up to 20% sequence divergence and overall sequence divergence of 3% (between *E. coli* phages M13 and fd DNA) slows *E. coli* RecA-mediated DNA formation of *jm* intermediates, and reduces *nc* product accumulation (DasGupta and Radding, 1982; Bianchi and Radding, 1983; Fabisiewicz and Worth, 2001).

**FIGURE 7:**
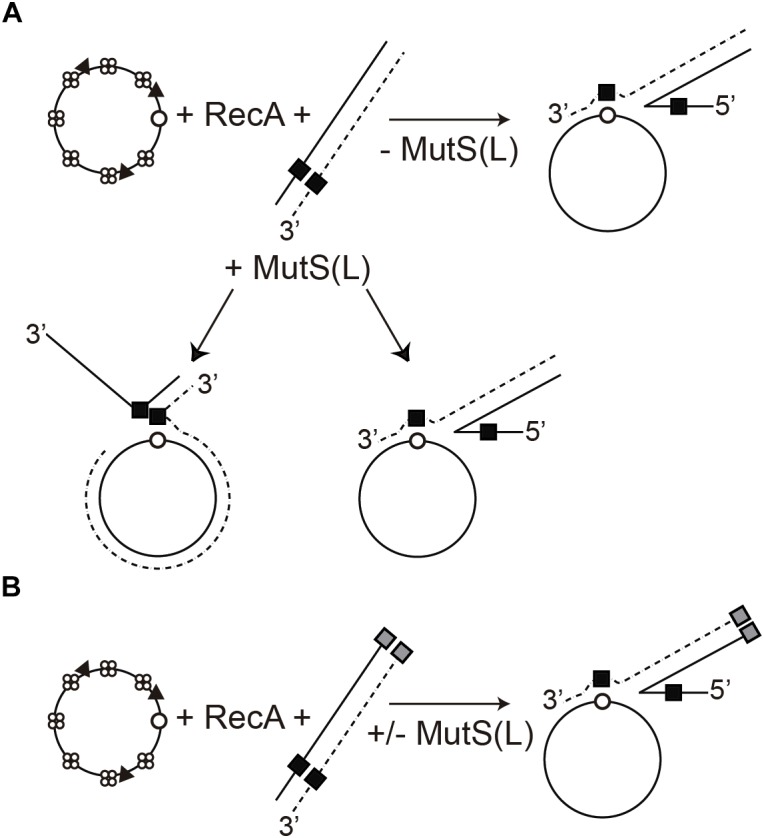
Model of the role of MutS(L) on RecA-mediated DNA strand exchange on heterologous substrates. **(A)** RecA-mediated recombination between *css_hom_* (open circle) DNA complexed with SsbA (four circles) and RecO (filled triangle) and the short heterologous segment (denoted by a black square) in the *lds_het_* DNA leads to *jm* intermediates and *nc* products in the presence of MutS(L), but only to *jm* in the absence of MutS(L). **(B)** Scheme of RecA-mediated recombination reaction between *css_hom_* and *lds_het–ins_* (black and gray squares) substrates, only leads to *jm* intermediates in the presence or absence of MutS(L). + and – denote the presence or absence of MutS(L).

Increasing *B. subtilis* MutS or MutL concentrations slightly reduce RecA⋅ATP-mediated DNA strand exchange with both homologous (*css_hom_* and *lds_hom_*) and homeologous (*css_hom_* and *lds_mis_*) DNA substrates (Figure 2B,C, lanes 2–4 vs. 1 and Supplementary Figure S3, lanes 3–5, 6–8 or 9–11 vs. 2), rather than RecA⋅dATP-mediated DNA strand exchange (Figure 2B,C, lanes 6–8 vs. 5). In contrast, *E. coli* MutS and MutL block RecA⋅ATP-mediated strand exchange in the presence of two discrete regions with up to 20% sequence divergence between phages M13 and fd DNA (Worth et al., 1994; Tham et al., 2013). It is likely that *B. subtilis* RecA-mediated bidirectional DNA strand exchange (Carrasco et al., 2016) could make MutS a modulator, rather than a barrier to recombination as shown for *E. coli* MutS and MutL on RecA-mediated strand exchange (Worth et al., 1994; Tham et al., 2013).

RecA⋅ATP-mediated DNA strand exchange preferentially initiated at the 3′ homologous end is mainly blocked at the single internal 77-bp segment with ∼54% heterology in otherwise homologous substrate (Figure 3A, lane 2 and Figure 4B,D [−MutS]). Similarly, *E. coli* RecA-mediated DNA strand exchange between viral ϕX174 vs. G4 DNAs, with an overall sequence divergence of 30% or with discrete segments with up to 55%, defining heterology, is blocked (DasGupta and Radding, 1982). The addition of MutS, however, apparently overcame the barrier between the DNA substrates (*css_hom_* and *lds_het_*) with a discrete heterologous segment (Figure 3A, lanes 3–5, Figure 3C, lanes 8–10 and 8′–10′, and Figure 4C,E [+MutS]). The biochemical properties of MutS suggest us a cellular role such as that depicted in Figure 7A. It is likely that MutS bound to the heterologous region could prevent further RecA filament growth from the recombination initiated at the 3′-end, rather than allowing RecA to bypass or reinitiate beyond the heterologous region to overcome the heterologous barrier (Figure 3B, 6–9 vs. 2–5). If the RecA filament cannot grow, RecA passively disassembles (Cox, 2003; Bell and Kowalczykowski, 2016). It is likely that free RecA can re-assemble at the 5′-end and re-initiate DNA strand exchange in the 3′→ 5′ direction, rather than removing the heterologous barrier (Figure 3C, lanes 8–10 and 8′–10′ and Figure 7A). To support this hypothesis a substrate with the 77-bp of internal heterology and also a heterologous 77-bp segment at the 5′-end was tested. Using the *css_hom_* and *lds_het–ins_* DNA substrates RecA-mediated strand exchange accumulates *jm* intermediates, but not *nc* products in the presence or absence of MutS (Figure 5, 6B–E, 7B). In other words, MutS might facilitate passive disassembly of RecA at the blocked recombination intermediate, but it cannot enable RecA-mediated recombination to overcome the heterologous barrier (Figure 7B).

The differences between interspecies conjugation (∼10^3^-fold increase in the *mutS* or *mutL* context with up to 16% sequence divergence) in γ-proteobacteria (Rayssiguier et al., 1989, 1991; Vulic et al., 1997) and the interspecies chromosomal transformation frequency (∼2-fold increase in the *mutSL* context with up to 15% sequence divergence) might lie in the physiological differences of the HGT mechanisms and the polarity of RecA-mediated DNA strand exchange. First, the incoming linear ssDNA in conjugation is soon converted into its duplex form (a step sensitive to mismatch correction) (Matic et al., 1996). Second, the conjugative linear dsDNA undergoes end processing to generate a 3′-tailed duplex, which determines the direction of RecA filament extension (Kowalczykowski et al., 1994). Third, *E. coli* RecA⋅ATP catalyzes unidirectional DNA strand exchange in a 5′→3′ direction (Cox and Lehman, 1981; West et al., 1981; Kowalczykowski, 1991; Konforti and Davis, 1992). Forth, *E. coli* MutSL by blocking RecA-mediated DNA strand exchange in a 5′→3′ direction (Worth et al., 1994; Tham et al., 2013) works as an anti-recombination mechanism (Matic et al., 1996). In contrast, during *B. subtilis* interspecies chromosomal transformation, RecA polymerizes on the incoming linear ssDNA, which shows no polarity at the entry site, followed by homology search with recipient duplex, and RecA-mediated bidirectional integration on the non-replicating haploid genome (steps insensitive to mismatch correction). When a homeologous segment is found, MutS bound to the heteroduplex region might impede RecA filament growth and indirectly might facilitate passive RecA disassembly. Free RecA might reengage with the distal 5′-end and catalyze DNA strand exchange in the 3′→5′ direction. It is observed that interspecies chromosomal transformation marginally decreases (∼2-fold) in the *wt* control when compared to the *mutSL* strain (Figure 1A), suggesting that a fraction of the homeologous substrate might be unproductive in the *wt* control, perhaps by some topological constrains if the RecA filament grown in the 5′→3′ direction is fully disassembly.

### Homology-Facilitated Illegitimate Recombination

Beyond 15% sequence divergence and up to 23% the frequency of interspecies chromosomal transformation increases 40- to 100-fold in the *mutS* or *mutSL*, when compared to the *wt* control (Figure 1A). An analysis of integrated DNA with ∼17% and ∼23% sequence divergence indicated that ∼63% and ∼93%, respectively, of the sequenced Rif^R^ colonies has a single *rpoB*482 mismatch that cannot be distinguished from a spontaneous mutation (a single *rpoB*482 point mutation). From the transformants with at least two mismatches (genuine transformants), the mean length of the integrated segment was ∼4-bp. Here, the length of the integrated segment was 8- to 12-fold below MEPS (Figure 1B). Based on data for RecA activities (Bell and Kowalczykowski, 2016), we considered unlikely that RecA could mediate DNA strand exchange of a ∼4-bp segment bearing two mismatches. This chromosomal transformation involving integration of very short segments (∼4-nt) requires RecA, because genuine transformants were not observed upon RecA inactivation.

Chromosomal transformation involving integration of very short segments (3- to 10-nt), via HFIR, has been documented in competent *S. pneumoniae* (Prudhomme et al., 2002), *A. baylyi* (de Vries and Wackernagel, 2002) and *P. stutzeri* cells (Meier and Wackernagel, 2003). Here, RecA during homology search might anchor a ssDNA region on the recipient substrate that via illegitimate recombination permits the integration of short linear ssDNA segments (Brigulla and Wackernagel, 2010). To accommodate all our data, we proposed that short stretches of microhomology between donor and recipient DNAs are anchored by RecA, and an undefined strand-annealing protein might catalyze HFIR. MutS or MutSL bound to the RecA heteroduplex might provide a barrier to RecA filament growth, and indirectly facilitate its disassembly, reducing HFIR. Indeed, integration of very short segments was blocked in Δ*recA* cells and increased ∼100-fold in the Δ*mutSL* strain when compared to the *wt* strain (Figure 1A). This increase, however, is an overestimation, because the transformation frequency was only less than threefold greater than the spontaneous Rif^R^ mutation rate in competent Δ*mutS* and Δ*mutSL* cells. We consider unlikely a single crossover integration of *rpoB*482 DNA or error-prone non-homologous end-joining reconnecting broken end because this event should disrupt the essential *rpoB* gene, leading to cell death. Alternatively, the incoming ssDNA must be able to co-align with recipient DNA and such recombination-independent pairing is provided by the persistent association of patches of microhomology at specific DNA structures (Overballe-Petersen et al., 2013). Our results exclude models involving G-quartets and DNA triplexes that require specific nucleotide sequences, such as poly-G and polypurine-polypyrimidine tracts surrounding the *rpoB*482 mutation. Many details of this rare hybrid transformation events remain to be explored.

## Author Contributions

JA conceived and supervised the study and wrote the manuscript. BC, ES, and JA designed research. BC and ES performed biochemical and AM-G and FM-H performed AFM experiments and their data analysis.

## Conflict of Interest Statement

The authors declare that the research was conducted in the absence of any commercial or financial relationships that could be construed as a potential conflict of interest.
