# Supplementary material for: Bacillus subtilis MutS Modulates RecA-Mediated DNA Strand Exchange Between Divergent DNA Sequences
Source: Front Microbiol. 2019 Feb 13;10:237. doi: 10.3389/fmicb.2019.00237 (PMC6382021; doi:10.3389/fmicb.2019.00237)
Supplement: Supplementary file 1 [file Presentation_1.pdf]

Supplementary Material

Bacillus subtilis **MutS modulates RecA-mediated DNA strand exchange between divergent DNA sequences**

Begoña Carrasco[1,†], Ester Serrano[1,†], Alejandro Martín-González[2], Fernando Moreno-Herrero[2] and Juan C. Alonso[1,*]

[1]Department of Microbial Biotechnology, Centro Nacional de Biotecnología, CNB-CSIC, 28049 Madrid, Spain.

[2]*Department of Macromolecular Structures, CNB-CSIC, 28049 Madrid, Spain*

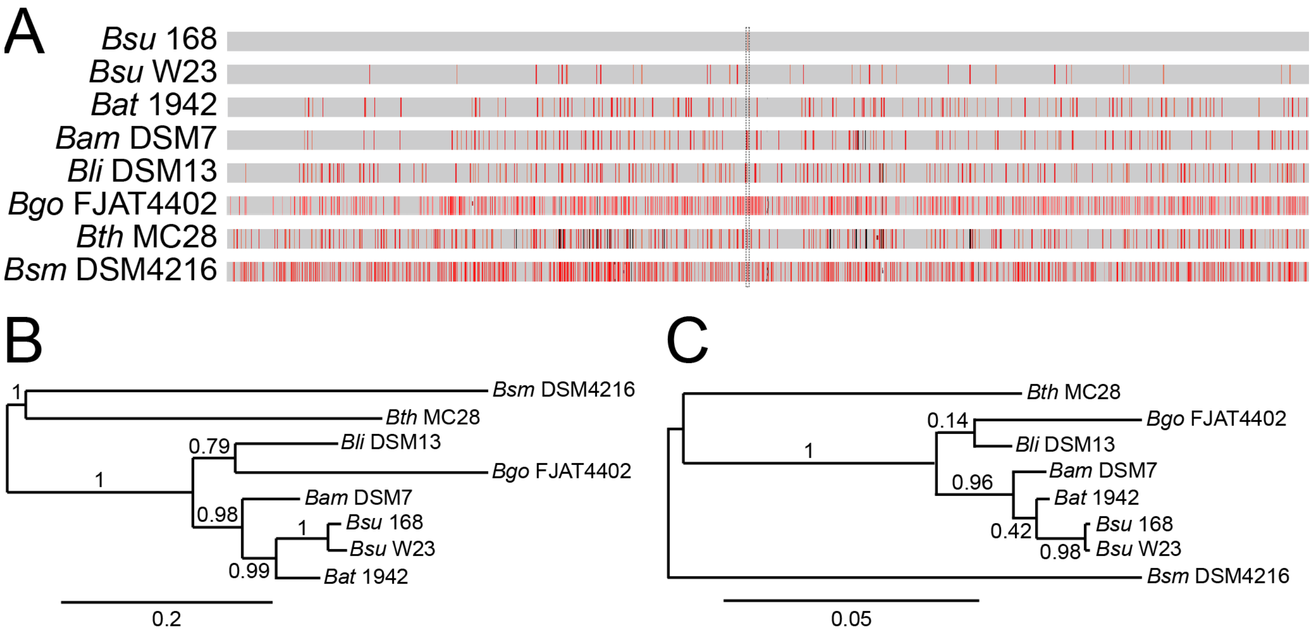

Supplementary Figure 1. Distribution of sequence divergence among different *Bacillus* species. (**A**) A single C-to-T transition mutation at codon 482 (framed by dotted lines) in the essential *rpoB* gene confers *Rif*^{R} (*rpoB*482 gene). The *rpoB*482 DNA was derived from *B. subtilis* 168 (*Bsu* 168), *B. subtilis* W23 (*Bsu* W23), *B. atrophaeus* 1942 (*Bat* 1942), *B. amyloliquefaciens* DSM7 (*Bam* DSM7), *B. licheniformis* DSM13 (*Bli* DSM13), *B. gobiensis* FJAT-4402 (*Bgo* FJAT4402), *B. thuringiensis* MC28 (*Bth* MC28) and *B. smithii* DSM4512 (*Bsm* DSM4216). Mismatches between Rif^R donor and the corresponding *rpoB* in the Rif^S recipient strain are indicated by vertical red bars, and insertions/deletions by vertical black bars. Bar thickness represents the number of mismatches in the neighborhood. (**B** and **C**) Phylogenetic tree of the selected *Bacillus* species or subspecies. The tree is based on the *rpoB*482 nucleotide sequence (**B**), and of the RpoB protein sequence (**C**). Branching confidence values are based on 1,000 bootstrap replicates. The alignment was performed using FASTA, BLAST, ClustalW, PhyML and MUSCLE 3.6.

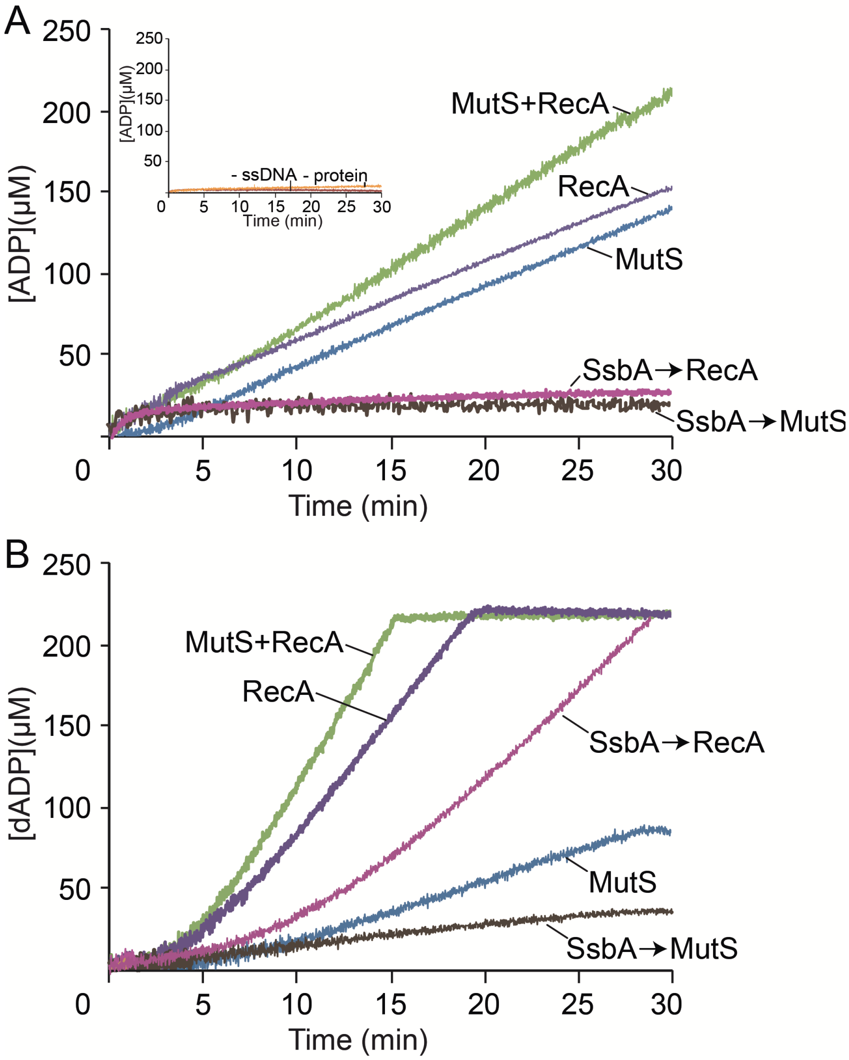

C

Proteins[a]	Condition	Lag time (in min)	k_{cat} min^{-1}
RecA (1 RecA/12-nt)	ATP[b]	< 1	8.6 ± 0.2
	dATP[b]	4 ± 0.5	17.9 ± 0.3
SsbA (SsbA/33-nt) + RecA	ATP[b]	NA	< 2
	dATP[b]	9 ± 0.4	16.6 ± 0.3
MutS (1 MutS/ 14-nt)	ATP	2 ± 0.3	7.1 ± 0.3
	dATP	2 ± 0.4	5.0 ± 0.7
SsbA (SsbA/33-nt) + MutS	ATP	NA	< 2
	dATP	NA	< 2
MutS + RecA	ATP	< 1	4.9 ± 0.2
	dATP	4 ± 0.4	13 ± 0.4

Supplementary Figure 2. MutS effects on RecA nucleation and polymerization onto ssDNA. The 3276-nt css_{hom} DNA (10 µM in nt) was incubated with MutS (0.7 µM), RecA (0.8 µM), or both MutS and RecA, or preformed SsbA-ssDNA complexes (SsbA, 0.3 µM) were incubated with MutS or RecA in buffer A containing 5 mM ATP (**A**) or dATP (**B**); the ATPase/dATPase activity was then measured (30 min, 37ºC). Inset in (**A**) is a control experiment in the absence of ssDNA or any RecA or MutS. (**C**) Lag times and maximal rate of nucleotide hydrolysis are shown for different experimental conditions. NA: Not applicable. Results shown as mean ± SEM of ≥3 independent experiments.

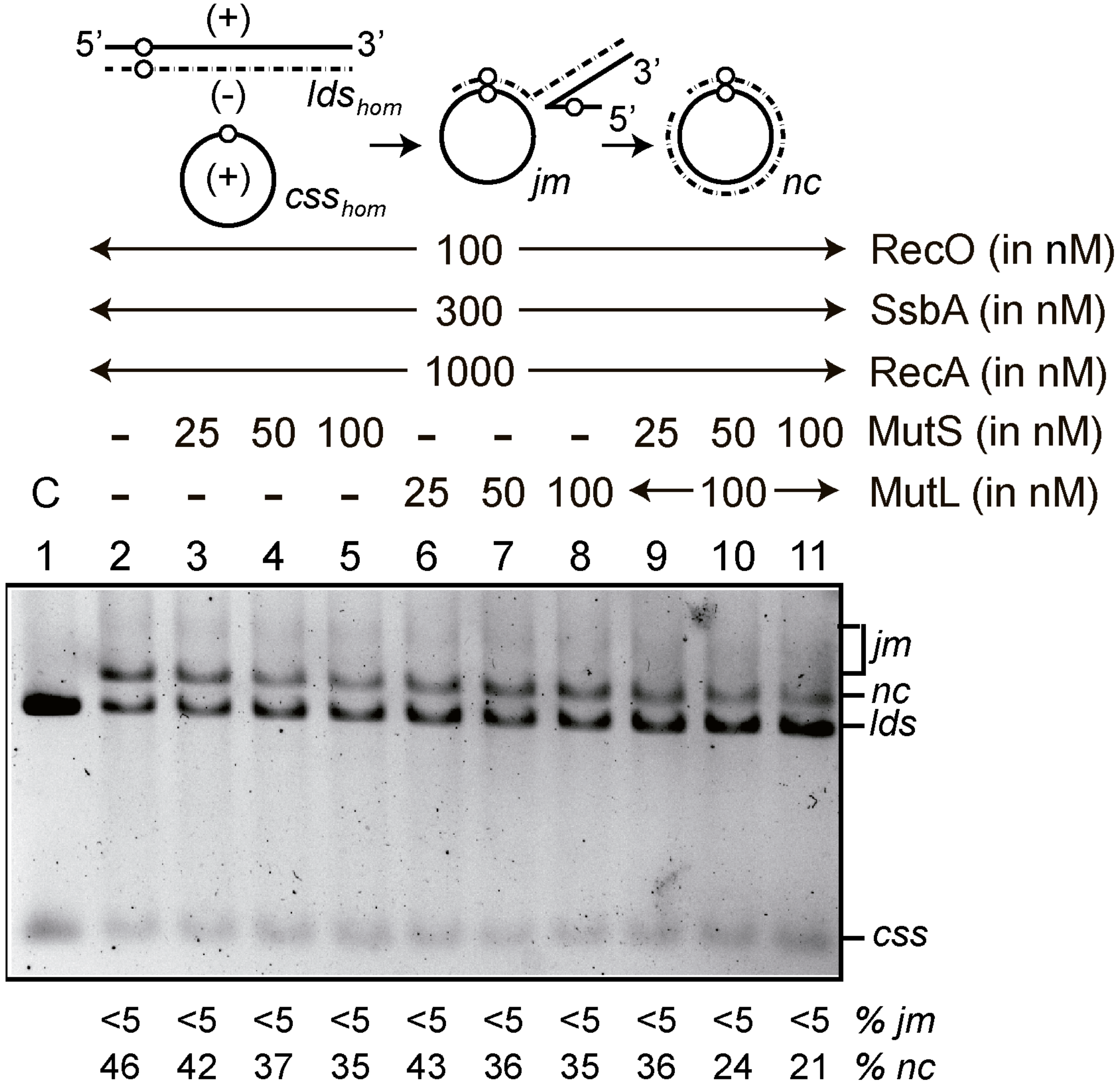

Supplementary Figure 3. RecA·ATP-mediated DNA strand exchange in the presence of MutSL. The scheme shows the three-strand exchange reaction between homologous css_{hom} (+ strand) and the homologous (- strand) lds_{hom} (open circle) substrates. The 3276-nt css_{hom} DNA (10 µM in nt) was preincubated with SsbA and RecO (5 min, 37°C) in buffer A containing 5 mM ATP, followed by RecA, (MutS, MutL or MutSL) and the 3276-bp lds_{hom} DNA (20 µM in nt) substrate. The reaction was incubated (60 min, 37ºC) and separated by 0.8% agarose gel electrophoresis. The positions of the bands corresponding to substrates (*css*, *lds*), intermediates (joint molecule, *jm*) and products (nicked circular, *nc*) are indicated. Lane 1, the *css* and *lds* substrates (termed C). The percentage of recombination intermediates (*jm*) and products (*nc*) are shown beneath the gel. Results shown as mean ± 5% SEM of ≥3 independent experiments. The minus (-) symbol denotes absence of MutS or MutL.

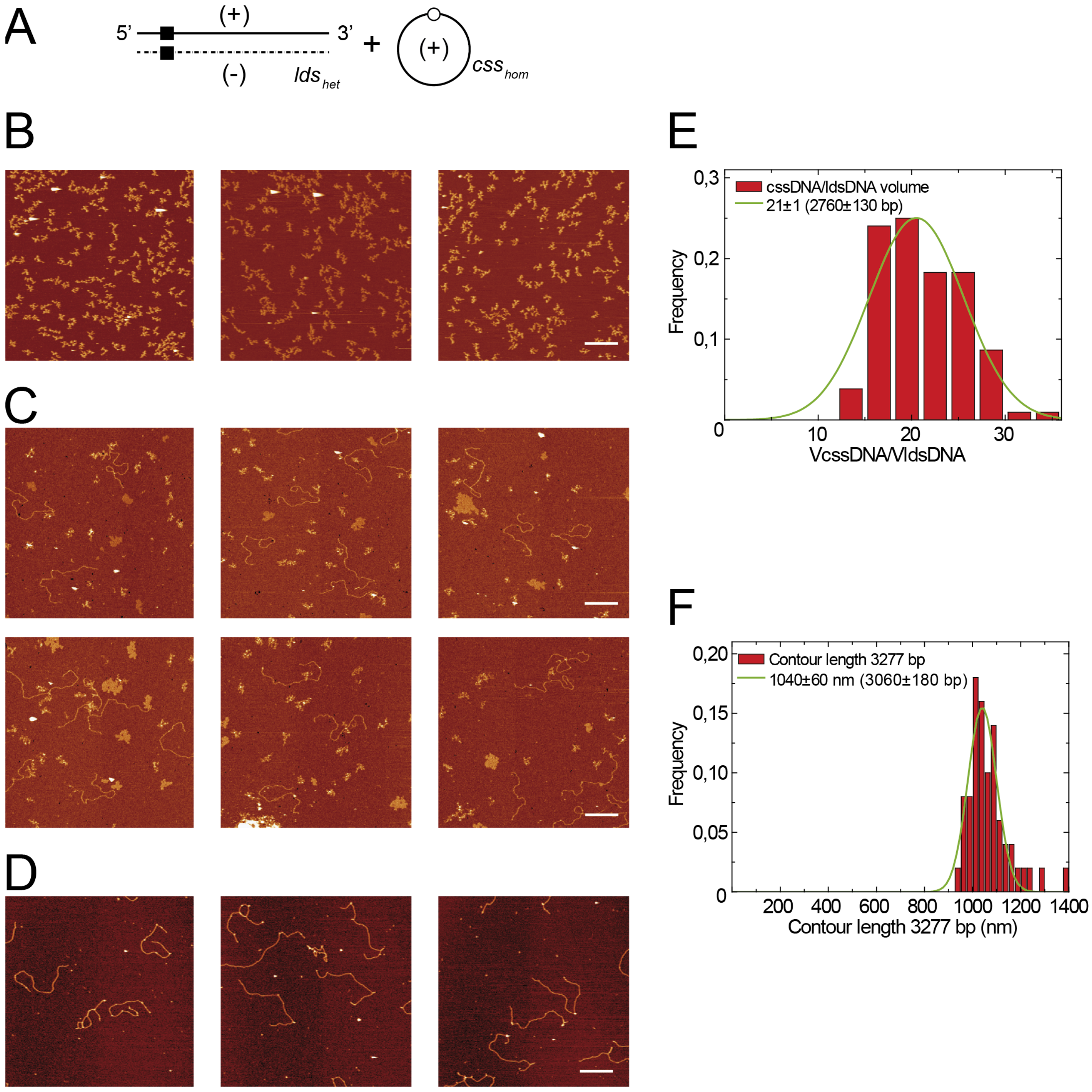

Supplementary Figure 4. AFM control experiments of lds_{het} and css_{hom} DNA. (**A**) drawing of the lds_{het} (black square) and css_{hom} (open circle) DNA substrates. DNA images of css_{hom} (**B**), css_{hom} and lds_{het} (**C**) and lds_{het} (**D**). The volume and the length histogram fitted to a Gaussian function for css_{hom} (**E**) and lds_{het} (**F**) are shown. Scale bar = 500 nm.
